# Holding it together: rapid evolution and positive selection in the synaptonemal complex of *Drosophila*

**DOI:** 10.1186/s12862-016-0670-8

**Published:** 2016-05-05

**Authors:** Lucas W. Hemmer, Justin P. Blumenstiel

**Affiliations:** Department of Ecology and Evolutionary Biology, University of Kansas, Lawrence, KS 66045 USA

**Keywords:** *Drosophila*, Synaptonemal complex, Positive selection

## Abstract

**Background:**

The synaptonemal complex (SC) is a highly conserved meiotic structure that functions to pair homologs and facilitate meiotic recombination in most eukaryotes. Five *Drosophila* SC proteins have been identified and localized within the complex: C(3)G, C(2)M, CONA, ORD, and the newly identified Corolla. The SC is required for meiotic recombination in *Drosophila* and absence of these proteins leads to reduced crossing over and chromosomal nondisjunction. Despite the conserved nature of the SC and the key role that these five proteins have in meiosis in *D. melanogaster*, they display little apparent sequence conservation outside the genus. To identify factors that explain this lack of apparent conservation, we performed a molecular evolutionary analysis of these genes across the *Drosophila* genus.

**Results:**

For the five SC components, gene sequence similarity declines rapidly with increasing phylogenetic distance and only ORD and C(2)M are identifiable outside of the *Drosophila* genus. SC gene sequences have a higher *dN/dS* (ω) rate ratio than the genome wide average and this can in part be explained by the action of positive selection in almost every SC component. Across the genus, there is significant variation in ω for each protein. It further appears that ω estimates for the five SC components are in accordance with their physical position within the SC. Components interacting with chromatin evolve slowest and components comprising the central elements evolve the most rapidly. Finally, using population genetic approaches, we demonstrate that positive selection on SC components is ongoing.

**Conclusions:**

SC components within *Drosophila* show little apparent sequence homology to those identified in other model organisms due to their rapid evolution. We propose that the *Drosophila* SC is evolving rapidly due to two combined effects. First, we propose that a high rate of evolution can be partly explained by low purifying selection on protein components whose function is to simply hold chromosomes together. We also propose that positive selection in the SC is driven by its sex-specificity combined with its role in facilitating both recombination and centromere clustering in the face of recurrent bouts of drive in female meiosis.

**Electronic supplementary material:**

The online version of this article (doi:10.1186/s12862-016-0670-8) contains supplementary material, which is available to authorized users.

## Background

In sexually reproducing eukaryotes, successful meiosis ensures faithful transmission of a haploid set of chromosomes to the next generation. Problems arising during meiosis can lead to meiotic arrest, chromosomal nondisjunction, and infertility. A key step in meiosis is the close alignment of homologous chromosomes, a process known as synapsis. Synapsis is typically essential for establishing meiotic crossovers and a specialized, tripartite protein structure known as the synaptonemal complex (SC) forms the foundation for synapsis [1–3].

The SC has been cytologically observed across eukaryotes and the molecular components have been characterized in a range of model organisms including *Arabidopsis thaliana*, *Caenorhabditis elegans, Drosophila melanogaster*, *Saccharomyces cerevisiae*, *Mus musculus*, and several species of *Hydra* [2, 4, 5]. Across this diverse group of eukaryotes the SC maintains, with some exceptions, evolutionary conservation in both structure as a tripartite complex and function in meiotic recombination and synapsis [2]. The SC consists of three main parts in most eukaryotes: the lateral elements (LEs), the transverse filaments (TFs) and the central element (CE) [6–8]. Two LEs run along the length of each pair of sister chromatids and directly interact with the meiotic cohesin complex. The TFs extend out from the LEs, resembling rungs of a ladder connecting the juxtaposed chromosomes. The CE is a solid visible element in the center of the TFs and secures them in place. Some eukaryotes lack an observable SC including *Schizosaccharomyces pombe* and *Aspergillus nidulans* [9–11]. In the case of *S. pombe*, the SC may have been replaced by thin thread-like structures known as the linear elements [12]. *D. melanogaster* males also lack the SC. This coincides with the fact that *D. melanogaster* males also have no meiotic recombination. These observations indicate that other mechanisms can ensure proper chromosome segregation in the absence of the SC.

Despite the strong structural conservation across eukaryotes, the proteins that comprise the SC are strikingly varied [13]. Based on the fact that several eukaryote lineages lack the SC [14–16], several authors have theorized that the SC has evolved independently multiple times [2, 4, 17]. However, a recent analysis [5, 18] found that *M. musculus* SC proteins formed monophyletic groups with orthologs in metazoans ranging from cnidarians to humans. This supports a hypothesis of a single SC origin in at least all metazoans. The SC of the Ecdysozoa (which includes molting animals such as crustaceans, *D. melanogaster*, and *C. elegans*) appears substantially different from the SC in other metazoans. SC components from species such as *D. melanogaster* and *C. elegans* show low conservation outside arthropods and nematodes, respectively. The reason for such lack of conservation of SC components is unknown [5, 18].

**Fig. 1:**
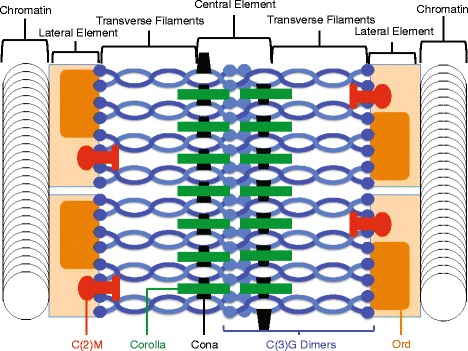
A model of the synaptonemal complex in *Drosophila.* This model is adapted from Lake & Hawley, 2012 [3] and Collins et al. 2014 [21]. So far five genes have been found coding SC components and their proteins localized within the structure: *ord*, *c(2)M*, *c(3)G*, *corolla*, and *cona*

Several SC proteins have been identified and characterized in *D. melanogaster*. Five such proteins are included in this study. EM studies in *D. melanogaster* females indicate the SC is similar in structure to other eukaryotes [1, 8] and all five proteins are contained within the tripartite structure [19–21] (Fig. 1). ORD and C(2)M have been identified as two of the LE proteins in *Drosophila* [20, 22, 23]. ORD localizes to the chromosome arms during early prophase I and is necessary for chromosome segregation, loading of the cohesin complex on the chromosomal axis, normal levels of meiotic recombination, and SC stability [20, 22, 24, 25]. Its role in crossing over is not entirely understood as recombination is not completely eliminated in *ord* mutants and there is an increased amount of DSB repair via the sister chromatid. This suggests that ORD suppresses sister chromatid exchange [20]. C(2)M is also a component of the LE and is responsible for chromosome core formation [25], SC-dependent meiotic DSB repair, and assembling a continuous CE [2, 23, 26]. The N-terminus of C(2)M lies within the inner region of the LE and the C-terminus is assumed to face the central region [26]. So far, C(3)G is the only known *Drosophila* TF protein [3]. Like other TF proteins, it has globular N- and C-terminal domains and an internal coiled-coil central domain [2]. C(3)G forms into parallel dimers with the N-terminal globular domains extending into the CE and the C-terminal domains are anchored to the LE [26]. C(3)G is necessary for synapsis, conversion of DSBs into crossovers [19, 27] and perhaps gene conversion [28]. Finally, the CE is comprised of two other proteins along with the C(3)G N-termini, Corona and Corolla. Corona, commonly referred to as CONA, is a pillar-like protein that aligns outside of the dense CE [3]. CONA promotes DSB maturation into crossovers and synapsis does not occur in *cona* mutants [29]. Additionally, CONA both co-localizes with C(3)G and stabilizes C(3)G polycomplexes [29]. Corolla is also localized within the CE and interacts with CONA [21]. Thought to be comprised of coiled-coil domains much like C(3)G, it is also essential for SC function and recombination. All of these proteins have roles exclusive to female meiosis except for ORD, which also functions in sister-chromatid cohesion in Meiosis I and II and is necessary for gametogenesis in both *Drosophila* sexes [30, 31].

Two hypotheses have been proposed to explain the lack of conservation of the SC: genetic drift and positive selection. A high rate of evolutionary drift in protein evolution in *Caenorhabditis* and *Drosophila* has been proposed to explain the evolution of the lamin proteins [32, 33] and ribosomal proteins in Ecdysozoa [34] as well as olfactory genes in *Drosophila* [35]. Low levels of purifying selection on *Drosophila* SC components would allow it to diverge at a high rate resulting in little conservation. Low levels of purifying selection might be expected if the major function of the SC was simply to hold homologs together at a proper distance. Under this scenario, there may be few selective constraints on the particular amino acids that function primarily as structural spacers within the SC.

Alternatively, positive selection may contribute to the rapid evolution of SC components. Many studies have demonstrated that reproductive proteins evolve rapidly [36–42]. In fact, population genetic analyses in *D. melanogaster* and close relatives have previously revealed that *ord* shows a significant deviation from neutrality in *D. simulans*, with more non-synonymous fixations than expected [42]. Recurrent meiotic drive and selection to ameliorate this conflict has been proposed to drive positive selection in meiosis genes [42–44].

We aimed to perform a molecular evolutionary analysis of the SC proteins in *Drosophila* to determine what forces may be driving the high rate of evolution of these proteins. Using the genomic sequence data available for different *Drosophila* species and *D. melanogaster* population data, we aimed to test the null hypothesis that divergence in SC proteins is effectively neutral. In addition, we sought to test the hypothesis that patterns of molecular evolution in SC components are uniform across the genus. Finally, we examined available *D. melanogaster* population data to determine if any deviations from neutrality have occurred in recent time, which would be consistent with ongoing positive selection.

## Methods

### Ortholog search

The amino acid sequences of *c(2)M* (CG8249; FBgn0028525), c*(3)G* (CG17604; FBgn0000246), *cona* (CG7676; FBgn0038612)*, corolla* (CG8316; FBgn0030852) and *ord* (CG3134; FBgn0003009) in *D. melanogaster* were acquired from FlyBase 5.57 [45]. An additional SC component, SOLO, was not examined due to the fact that it is an alternative splice variant of *vasa*, which is known to play a role in piRNA biogenesis [46]. These were used in a tBLASTn [47] homolog search in 21 available genomes of *Drosophila* species with a liberal cutoff of E = 0.1. This liberal cutoff was chosen to ensure detection of highly divergent orthologs that were subjected to further validation. *D. melanogaster*, *D. sechellia, D. yakuba*, *D. erecta, D. ananassae*, *D. pseudoobscura*, *D. willistoni*, *D. virilis*, *D. mojavensis,* and *D. grimshawi* genomes were obtained from FlyBase [45]. The genomes for *D. ficusphila* [GenBank: AFFG00000000.1]*, D. eugracilis* [GenBank: AFPQ00000000.1]*, D. biarmipes* [GenBank: AFPQ00000000.1]*, D. takahashii* [GenBank: AFFD00000000.1]*, D. elegans* [GenBank: AFFI00000000.1]*, D. bipectinata* [GenBank: AFFF00000000.1]*,* and *D. miranda* [GenBank: AJMI00000000.1] were obtained from NCBI. The genome of *D. simulans* was obtained from the Andolfatto lab server [48] and the *D. mauritiana* genome was obtained from the Schlötterer lab server [49]. To identify highly divergent orthologs, an additional tBLASTn search was performed using the most diverged protein sequence captured in the original tBLASTn search. These results were combined with results from BLASTp searches of annotated proteins using the *D. melanogaster* protein sequence. Finally, we included additional ortholog searches with HMMER 3.1b2 [50] and PhylomeDB v3 [51] as well as orthologs listed in OrthoDB v7 [52]. This combined approach allowed us to obtain a broad list of candidate orthologs for each of the five SC components. Orthology was then evaluated for candidates by using a reciprocal best BLAST hits approach with tBLASTn. In all cases where orthology was determined the second reciprocal BLAST hit E-value was substantially worse than the ortholog E-value. In addition, synteny for orthologs was evaluated (Additional file 1: Table S1), though it should be noted that there is substantial gene shuffling within Muller elements across the genus [53].

### Sequence retrieval

Upon identification of orthologs, sequences from annotated and un-annotated genomes were extracted using identical approaches to limit biases that might arise from using gene annotations only from annotated genomes. DNA sequences 3000 bp upstream and downstream of identified orthologous sequences were first extracted. These were analyzed with FGENESH+, a Hidden Markov Model protein-based gene predictor used to identify the open reading frames in un-annotated DNA sequence using a known protein sequence as a guide [54]. We included 3000 nucleotides of upstream and downstream flanking sequence to ensure that parts of the open reading frame not originally identified in tBLASTn were included. The *D. melanogaster* amino acid sequence was used as the guide.

### Sequence alignments and *Drosophila* phylogeny

Sequence alignments were generated using coding sequences (when identified) obtained with FGENESH+ from each species using both translational MAFFT [55] and translational MUSCLE [56] in Geneious v5.6 [57] with default parameters. Sequence alignments were also generated using codon-based PRANK [58] based on a pre-determined phylogenetic tree (see below) with the “-F” option allowing insertions. These three alignment programs were used to evaluate sensitivity of results to alignment procedure. Concatenated alignments of SC sequences (obtained either by MUSCLE or MAFFT) were used to generate phylogenetic trees required for PRANK alignment and other analyses. Phylogenetic analysis was performed using the Cipres Science Gateway v3.0 with RAxML-HPC Blackbox using default parameters and a GTR model with 100 bootstrap iterations [59]. The tree topologies produced by concatenated MAFFT and MUSCLE alignments were identical to each other. The SC gene tree topology also matched the known phylogeny for the *Drosophila* species used in this analysis [53].

### Molecular evolutionary analysis

The global omega (ω) value, often referred to as the global *dN/dS* estimate, is a measure of the average selective pressure acting on a gene across an entire phylogeny [60]. Global ω for each alignment was calculated using HyPhy with a GTR model [61] and also with the one-ratio model F3x4 codon model (M0) in the *codeml* program of PAML v4.4 [62]. Both analyses made use of the tree topology obtained from phylogenetic analysis described above. Global ω estimates were obtained using all available orthologs, a smaller subset of 12 species within the *melanogaster* group (*D. melanogaster*, *D. sechellia, D. simulans, D. mauritiana, D. yakuba*, *D. erecta, D. ficusphila, D. eugracilis, D. biarmipes, D. takahashii, D. elegans,* and *D. bipectinata*), and an even smaller subset of six species within the *melanogaster* subgroup (*D. melanogaster*, *D. sechellia, D. simulans, D. mauritiana, D. yakuba*, and *D. erecta)*. Estimates were obtained at different levels of divergence to account for potential problems that might occur in the alignment of highly diverged protein sequence.

To quantify heterogeneity in selection pressure, alignments were analyzed with GA Branch using a GTR model of nucleotide substitution [63] and the previously described phylogenetic tree. Analysis was performed using Datamonkey, the HyPhy web server [64]. GA Branch uses a genetic algorithm and the Akaike Information Criterion to identify the best fitting model for the number of branch ω classes. This allows one to evaluate evidence for heterogeneity in ω across the tree. A model-averaged probability of positive selection (ω > 1) on any of these branches is used to test whether positive selection has occurred.

An analysis of ω was also performed in PAML [62] by comparing two different codon based models of evolution. A likelihood ratio-test was performed to compare a model allowing a beta-distributed value of global ω ranging from zero to one (M7) to a model that also included an additional class of codons with ω greater than one (M8). Both of these models were run with the F3xF4 codon model using the nucleotide frequencies at each codon separately and the phylogenetic tree constructed above.

### Tests of neutrality using polymorphism and divergence

While codon models of molecular evolution provide insight into long-term patterns of selection acting on protein coding sequence, population genetic analyses allow for tests of neutrality in more recent time. McDonald-Kreitman (MK) tests of neutrality were performed using polymorphism data from two *D. melanogaster* populations and *D. simulans* and *D. yakuba* reference genomes served as outgroups. The Drosophila Genetic Reference Panel v1 (DGRP) [65] provided DNA sequences from 162 *D. melanogaster* isofemale lines collected from a population in Raleigh, North Carolina. In addition, 139 genomes from the Drosophila Population Genomics Project v2 (DPGP) [66] from 20 separate populations in Sub-Sahara Africa were used. SC gene sequences were collected using BLAST with *D. melanogaster* reference genes as the query. BLAST was performed locally in Geneious. Gaps in the alignment were removed and MK tests were performed online with the standardized and generalized MK test website [67]. Polarized MK tests were also performed using *D. yakuba* sequences to polarize lineage-specific substitutions. In addition, GammaMap [68] was used to identify particular codons within the SC genes of *D. melanogaster* that have likely been fixed by positive selection. A challenge of the MK test is that polymorphic sites are treated equally and allele frequencies are not taken into account. In contrast, GammaMap utilizes population and divergence data fully. Under a codon model of evolution, polymorphism and divergence data are used to estimate the distribution of fitness effects (DFE) for new mutations and substitutions. GammaMap estimates the γ parameter for each codon along the length of the gene. γ is the population-scaled selection coefficient, γ *=* 2*PN*_*e*_*s*, where *P* is the ploidy level, *N*_*e*_ is the effective population size, and *s* is the fitness advantage of a derived allele relative to the ancestral allele if the derived amino acid differs from the ancestral allele. Evidence for positive selection driving an amino acid substitution in *D. melanogaster* was deemed significant if the probability of γ greater than 0 was greater than 0.5 in *D. melanogaster* following Wilson et al. (2011). In addition, DnaSP 5.10.1 [69] was used to estimate average pairwise differences within each gene (π) and we compared these to the average pairwise site differences for other meiosis genes previously measured [42]. Tajima’s D was also calculated in DnaSP [70]. Haplotype structure was illustrated with phylogenetic trees built using UPGMA, a hierarchal clustering method [71], in Geneious 5.6.5 [57].

## Results

### Distant orthologs of drosophila SC components are elusive using diverse search methods

We assembled a list of candidate orthologs of SC components in *D. melanogaster* using BLAST, the HMMER search tool [50], and by consulting databases of listed orthologous genes including PhylomeDB and OrthoDB (Additional file 1: Table S2–S7). Orthologs were validated using the reciprocal best BLAST hit approach and hits were consistent with prior ortholog annotations. Only *c(2)M* and *ord* orthologs could be identified in all *Drosophila* species and further outside the genus (Additional file 1: Table S2–S7). The LE gene sequences were identified in every *Drosophila* species by tBLASTn and in several closely related Diptera species using BLASTp against annotated proteins (Additional file 1: Table S3 and S4). These include *Bactrocera cucurbitae* (melon fly), *B. dorsilas* (oriental fruit fly), *Ceratitis capitata* (Mediterranean fruit fly), *Musca domestica* (housefly) and *Glossina morsitans morsitans* (Tsetse fly). The remaining three SC components, *c(3)G*, *corolla*, and *cona*, could be identified in all species of *Drosophila* with annotated genomes using BLASTp (Additional file 1: Table S5–S7). The one exception is that *cona* could not be identified within *D. willistoni* (Additional file 1: Table S7). None of the TF and CE gene sequences could be identified outside of the *Drosophila* genus. These results suggest that the TF and CE proteins are less conserved than those comprising the LE.

### SC genes are evolving quickly and according to position within the SC

**Fig. 2:**
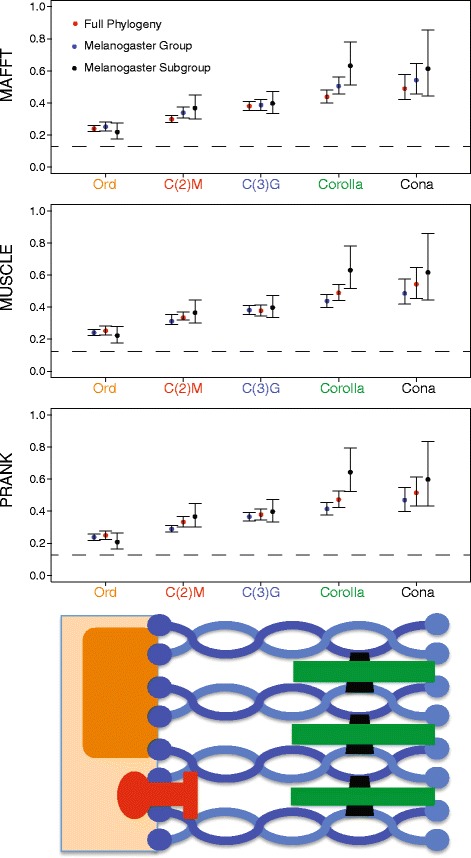
The ω ratio of each SC gene is high and robust to alignment and divergence level. ω was estimated in PAML using a GTR nucleotide substitution model with 95 % confidence intervals. The ratio remains relatively consistent for each alignment program used (MAFFT, MUSCLE, and PRANK) and the sampling from the phylogeny. ω estimates are reflected in spatial orientation of the protein within the complex respective to the chromosome; ω increases as distance from the chromosome increases. The dotted line indicates the median ω of odorant binding genes, the GO term with the largest median ω, from the 12 Drosophila Genomes Consortium [53]

HyPhy and PAML were used to calculate global ω with sequences obtained from the tBLASTn search. Orthologs that were only identified with BLASTp could not be reasonably aligned. Thus, the orthologs of *c(3)G* in *D. willistoni* and the *Drosophila* subgenus and orthologs of *cona* in *D. ananassae*, *D. bipectinata*, *D. willistoni*, and the *Drosophila* subgenus were not included in the molecular evolutionary analyses (Additional file 1: Tables S5 & S7). To account for possible issues with alignment quality for divergent sequences, we generated alignments with MAFFT, MUSCLE, and PRANK. The global ω estimates were robust to the three alignment methods (Fig. 2, Additional file 1: Figure S1). To account for long divergence times between many of the *Drosophila* species, global ω was also estimated across three different scales of divergence. We selected a subset of 12 species within the *melanogaster* group (*D. melanogaster*, *D. sechellia, D. simulans, D. mauritiana, D. yakuba*, *D. erecta, D. ficusphila, D. eugracilis, D. biarmipes, D. takahashii, D. elegans,* and *D. bipectinata*) and an even smaller set of six species within the melanogaster subgroup (*D. melanogaster*, *D. sechellia, D. simulans, D. mauritiana, D. yakuba*, and *D. erecta*). Global ω estimates were similar across different scales of divergence and different alignment methods (Fig. 2). The global ω of each SC component was higher than the median ω for each Gene Ontology (GO) category in *Drosophila* [53]. The majority of genes within *Drosophila* have ω estimates less than 0.1 [72] and only two GO categories have a median ω greater than 0.1 (response to biotic stimulus and odorant binding) [53]. *ord* has the lowest ω amongst all the SC genes at ~ 0.24 which is twice as high as the median ω for odorant binding genes and greater than the reported value for seminal fluid proteins (0.17) in the *D. melanogaster* species group [72].

There is an apparent relationship between position within the SC and ω. Although the LE component *ord* is evolving at more than twice the average genome-wide rate ratio, it has the lowest value of ω in the SC (ω: ~ 0.240 PAML, Fig. 2, ~ 0.265 HyPhy, Additional file 1: Figure S1). *cona* is evolving with the highest rate ratio (ω: ~ 0.500 PAML, Fig. 2, ~ 0.520 HyPhy, Additional file 1: Figure S1) and the global ω estimate is even higher within the species of the *melanogaster* subgroup (~0.600, Fig. 2, Additional file 1: Figure S1). The estimates of ω increase as a function of position within the SC: lateral element components evolve the slowest, central element components evolve the fastest, and *c(3)G,* which functions as a transverse filament, evolves at an intermediate rate. Because we have only characterized five proteins, there is little power in a test for significance in this relationship. However, it is worth noting that this result is robust to different time scales of analysis.

### Evolutionary rate ratio variation and signatures of positive selection

**Fig. 3:**
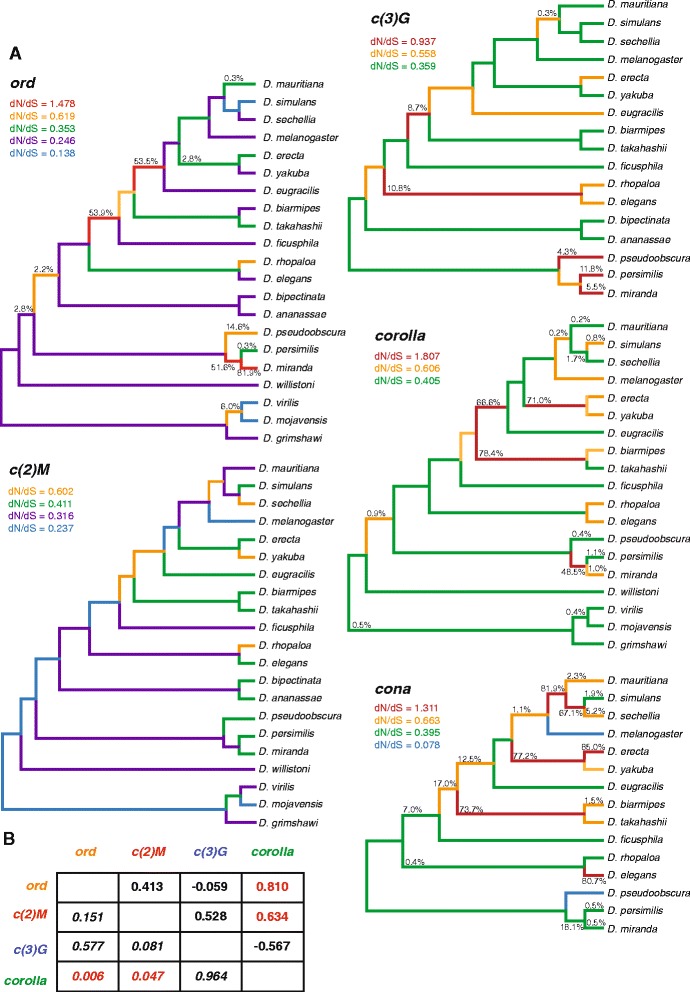
GA Branch analysis of the *Drosophila* phylogenetic tree reveals heterogeneity of evolutionary rates for each SC gene. **a** Supported rate ratio classes correspond to branch colors. Numbers on the branches present the posterior probability that a gene has evolved under positive selection along that particular branch. The phylogenetic trees correspond to the sequences of *ord*, *c(2)M*, *c(3)G*, *corolla*, and *cona* used in the molecular evolution analyses. **b** Evolutionary Rate Covariation analysis. Evolutionary Rate Covariation values (ERC) are above the diagonal. Values closer to 1 indicate higher levels of covariation. *P*-values are below the diagonal

We further tested for heterogeneity in ω estimates across the genus. GA Branch [63] uses a genetic algorithm to estimate and evaluate evidence for multiple classes of ω within a phylogenetic context using the Akaike Information Criteria. It further tests a model for averaged probability for ω > 1 for each branch. Results from GA Branch indicate that the evolutionary rate of SC components has varied considerably (Fig. 3a, Additional file 1: Figure S2 and S3). *c(3)G* and *corolla* have the fewest evolutionary rate ratio classes (three), *ord* had the most (five), and *c(2)M* and *cona* both have four rate ratio classes (Fig. 3a). There was support for positive selection (ω > 1) on at least one branch in every SC-coding gene except *c(2)M. corolla* had the highest ω estimate in any of the GA Branch analyses. *corolla* also demonstrated a strong signature of positive selection on the branch containing *D. biarmipes* and *D. takahashii* and also the branch prior to the split between *D. eugracilis* and the *melanogaster* subgroup (Fig. 3a). *cona* shows the most branches with signatures of positive selection (six). The LE protein *ord* has the lowest global ω but shows multiple branches with high probabilities of positive selection within the *obscura* group and prior to the *D. eugracilis* and *melanogaster* subgroup divergence. Along with the fact that *ord* had the most ω rate classes, this suggests that the evolution of *ord* is highly variable even amongst SC components. It should be noted that since alignment of divergent sequences can be challenging, ω estimates on deep internal branches might not be precise. However, rate ratio variation and significant evidence for positive selection are clearly evident on terminal branches. In particular, for each gene, support for the highest ω class on the phylogeny is evident on at least one terminal or near terminal branch.

Given this rate ratio heterogeneity, we sought to evaluate whether changes in ω estimates tended to co-occur among SC components. This would be the case if structural changes in one SC component drove structural changes in other SC components. A simple test for a correlation between branch ω estimates of different SC components must control for shared demographic changes that influence all proteins in the genome. Therefore, we employed the method of Evolutionary Rate Covariation [73, 74]. Clear, alignable orthologs of *cona* are found in the fewest number of species and *cona* was not included in this analysis, limiting this analysis to four SC components. We find significant evidence that ω estimates are correlated between *ord* and *corolla* and also *ord* and *c(2)M* (Fig. 3b). *c(3)G* shows no significant evidence of evolutionary rate co-variation with any other component, even though it interacts with both the lateral element and the central element.

Evidence for positive selection across the genus was evaluated using the M7 vs. M8 test in PAML. Two models of evolution were compared using a likelihood ratio test; a model with beta-distributed ω values less than one (M7) and the same model with an additional class of codons with ω values greater than one (M8) [62]. A significant likelihood test indicates a signature of positive selection. Positive selection is evident in *corolla* and this result is robust to both alignment procedure and sampling across different levels of divergence (Table 1). GA Branch also identified at least one branch with evidence of positive selection within each of the three levels of divergence. *c(3)G* also demonstrated evidence for positive selection within the *Drosophila* genus and *melanogaster* group but none was detected within the six species in the *melanogaster* subgroup. This is consistent with results from GA Branch that only identified branches with ω estimates near one outside of this clade. In contrast, *ord* showed significant evidence for positive selection in the *melanogaster* subgroup and nowhere else. The likelihood ratio tests and GA Branch both suggest that while *ord* is the most conserved of the SC components, positive selection intermittently contributes to its divergence. No signatures of positive selection were detected in *c(2)M* and *cona*. For *c(2)M*, this is consistent with results from GA Branch. However, the failure to reject a model of neutral evolution in *cona* stands in contrast to the positive selection detected on multiple branches by GA Branch. This may be explained by the fact that the *cona* coding sequence is much shorter and multiple branches were identified to be very conserved in GA branch. Under these circumstances, global PAML analysis of *cona* may have reduced power to detect a class of codons with ω greater than one.

**Table 1:** *P*-values of a likelihood ratio test between a model of variable selection pressures with no positive selection (M7) and the same model with positive selection (M8) for each SC component^*^

Gene & Alignment	Full Phylogeny	*mel* Group	*mel* Subgroup
ORD MAFFT	0.98	0.77	**7.9E-03**
ORD MUSCLE	0.38	1.00	**5.5E-03**
ORD PRANK	0.36	0.81	**4.9E-02**
C(2)M MAFFT	0.27	0.10	0.07
C(2)M MUSCLE	**0.02**	0.11	0.32
C(2)M PRANK	0.12	0.10	0.45
C(3)G MAFFT	**7.3E-07**	**2.5E-03**	1.00
C(3)G MUSCLE	**6.0E-16**	**1.7E-03**	1.00
C(3)G PRANK	**7.6E-03**	**0.02**	1.00
Corolla MAFFT	**1.2E-07**	**5.3E-08**	**1.3E-03**
Corolla MUSCLE	**6.5E-09**	**1.1E-07**	**0.03**
Corolla PRANK	**1.3E-04**	**5.3E-03**	0.05
CONA MAFFT	0.08	0.05	0.26
CONA MUSCLE	0.06	0.11	0.32
CONA PRANK	0.13	0.11	0.34

^*^Significant *P*-values in bold

The results of GA Branch and PAML complement each other and detect positive selection in most of the SC components. Both agree that *c(2)M* shows no sign of positive selection anywhere in the phylogeny or across different divergence times. The TF protein *c(3)G* does show signatures of positive selection outside of the *melanogaster* subgroup in both tests. Likewise, *corolla* shows evidence of positive selection throughout the *Drosophila* phylogeny across different time scales of divergence. Despite having the lowest calculated ω, *ord* shows strong a signature of positive selection within the *melanogaster* subgroup.

### Polymorphism and divergence in the D. melanogaster subgroup

To characterize the forces that have shaped the evolution of SC components in more recent time, we turn to readily available population data for *D. melanogaster*. We used the second Drosophila Population Genomics Project African survey of 139 genomes from 20 African *D. melanogaster* populations [66] as well as 162 genomes made available by the Drosophila Genetic Reference Panel, a sampling of inbred lines from Raleigh, North Carolina [65]. We performed a series of McDonald-Kreitman (MK) tests [75] using *D. simulans* sequences as an outgroup to test neutrality in divergence of SC components. To account for deleterious recessive polymorphisms that are retained at low frequencies, we removed singletons, doubletons, and tripletons. Additionally, the MK test can be used to calculate an alpha parameter – the proportion of substitutions that are positively selected [76]. A negative alpha value indicates the fixation or segregation of deleterious mutations within the gene. Polarized MK tests were also performed with the *D. yakuba* sequence as an outgroup.

The MK test revealed evidence for deviation from neutrality in some, but not all, SC components. Using population genetic data from *D. melanogaster* and *D. simulans* as an outgroup, an unpolarized MK test does not localize signatures of deviation from neutrality to a certain branch. Polarizing fixations on the *D. melanogaster* branch with *D. yakuba* as an additional outgroup allows one to determine whether the deviation from neutrality occurred on the *D. melanogaster* lineage. Across all tests, we find no evidence for recent selection in *c(2)M* and *cona* (Table 2), consistent with molecular evolutionary analyses. In contrast to its overall slowest ω estimate, but consistent with PAML results in the *D. melanogaster* subgroup (Table 1), *ord* is the only gene found to deviate from neutrality in both the polarized and unpolarized MK tests (Table 2), supporting previous results [42]. Positive alpha values from the polarized MK test indicate recent positive selection in *D. melanogaster*. Evidence for positive selection was found for *c(3)G* and *cona* in the unpolarized test using African populations only. However, polarized tests that examine fixations on the *D. melanogaster* lineage fail to reject neutrality for *c(3)G* and *cona*. Thus, the signature of positive selection in *c(3)G* and *cona* can be attributed to changes on the *D. simulans* lineage.

**Table 2:** McDonald-Kreitman tests (MKT) detecting deviation from neutrality within two population samples of *D. melanogaster* for all SC components^*^

		Unpolarized MKT^a^		Polarized MKT^b^	
		N. Carolina	Africa	N. Carolina	Africa
*ord*	α	**0.685**	**0.570**	**0.692**	0.581
	*p*	**0.021**	**0.042**	**0.034**	0.070
*c(2)M*	α	0.232	0.733	−0.007	0.657
	*p*	0.515	0.088	0.988	0.186
*c(3)G*	α	0.301	**0.709**	−0.111	0.511
	*p*	0.453	**0.005**	0.834	0.139
*corolla*	α	0.119	0.471	−0.036	0.371
	*p*	0.867	0.294	0.964	0.465
*cona*	α	0.450	**0.833**	−0.406	0.532
	*p*	0.429	**0.006**	0.680	0.321

^*^Significant *P*-values in bold

^a^Detects deviation within *D. melanogaster* and *D. simulans*

^b^Detects deviation within *D. melanogaster* exclusively

Further investigation revealed *D. simulans* was more highly divergent when compared to both *D. melanogaster* in four SC components (Table 3), with *ord* being the exception. *c(3)G* and *cona* both show an excess of non-synonymous divergence within *D. simulans* (Table 3). Thus, the results of the MK tests for *c(3)G* and *cona* can be explained by an excess level of non-synonymous divergence on the *D. simulans* lineage. This observation is also made in the GA branch analysis (Fig. 3a). Though not significant, both *c(2)M* and *cona* show a similar pattern of increased non-synonymous divergence in *D. simulans*. Pooling polarized fixations in every SC gene revealed significantly more non-synonymous fixations in *D. simulans* than *D. melanogaster* (2×2 *χ*^2^, N. Carolina *P* = 0.004, Africa *P* = 0.003).

**Table 3:** Fisher’s Exact tests reveal an increase of non-synonymous substitutions on the *D. simulans* lineage*

		N. Carolina			Africa		
	Substitution	*D. mel*	*D. sim*	*P*-value	*D. mel*	*D. sim*	*P*-value
*ord*	Non-syn	15	15	1	15	15	1
	Syn	20	21		20	21	
*c(2)M*	Non-syn	27	32	0.192	30	32	0.202
	Syn	33	23		36	23	
*c(3)G*	Non-syn	36	47	**0.030**	31	47	**0.025**
	Syn	40	24		36	24	
*corolla*	Non-syn	37	35	0.544	35	33	0.539
	Syn	23	15		22	15	
*cona*	Non-syn	8	17	**0.032**	7	17	**0.014**
	Syn	9	3		9	3	
Total	Non-syn	123	146	**0.004**	118	144	**0.003**
	Syn	125	86		123	86	

*Significant *P*-values in bold

The MK test is inadequate for identifying the codons that have been fixed positive selection. We therefore complemented the MK approach using GammaMap [68] to estimate the γ selection coefficient for each codon. Similar to the MK test, GammaMap utilizes both polymorphism and divergence data. However, it also makes use of frequency data to estimate the strength of selection that has acted individual codons. The selection coefficient is expressed in terms of γ, which is equal to *2PN*_*e*_*s*, twice the product of the effect population size multiplied by the ploidy level and the selection coefficient. In accordance to Wilson et al. 2011 [68], we used the probability of γ > 0 being 50 % or greater as a cutoff for a significant signature of positive selection [68]. Since we were using polymorphism data from *D. melanogaster*, we did not perform estimation of γ in *D. simulans*.

**Fig. 4:**
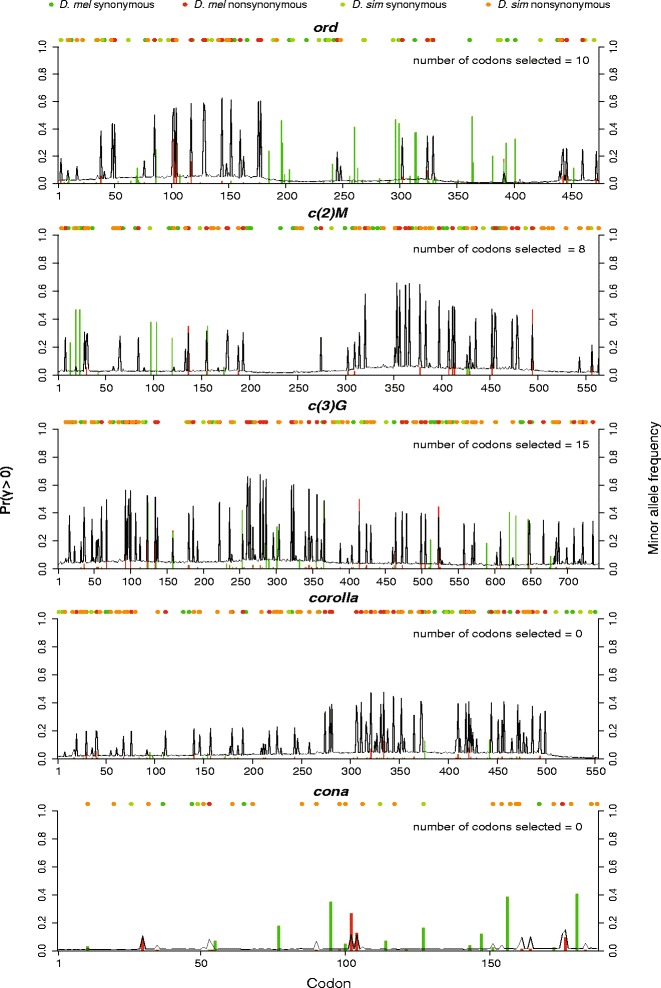
GammaMap reveals the posterior probability for positive selection coefficient at each codon using population data from the DPGP. In concordance with Wilson et al. 2011, a codon is under significant signature of selection when the posterior probabilities of selection (*lines*) are greater than 0.5 (primary Y-axis). Vertical bars illustrate minor allele frequencies in *D. melanogaster* (secondary Y-axis) and the substitutions are the circular dots. The colors correspond to *D. melanogaster* non-synonymous (*red*) and synonymous (*dark green*) variants as well as *D. simulans* non-synonymous (*orange*) and synonymous (*light green*) variants. Estimated number of selected codons is indicated in the upper right of each plot

Overall, signatures of positive selection on the *D. melanogaster* lineage are demonstrated for all SC proteins across the entire length, with the exception of *cona*. The distribution of putative selection effects were similar using data from two subpopulations of *D. melanogaster* (Fig. 4, Additional file 1: Figure S4), though more codon variants were deemed significant for evidence of positively selection using data from the North American subpopulations compared to African populations. For example, results for *corolla* using African data provide no significant evidence for recent positive selection at the 50 % threshold, in contrast to results using North American data. This is likely an effect of recent demographic history in North America [77–80]. Additionally, *corolla* sequences contain many low-frequency segregating alleles that are potentially deleterious. Using DGRP data, no codons in *c(2)M* were identified to be under significant positive selection while there were six noted in using population data from Africa (Fig. 4, Additional file 1: Figure S4). Overall, many of the same codons estimated to be putatively positively selected using data from one population were also were also found using data from the other population. *ord* and *corolla* show evidence of weak positive selection in specific regions, specifically between codons 50 and 200 in *ord* and between codons 300 and 500 in *corolla* (Fig. 4). Evidence for selection was also concentrated in *c(2)M* between codons 350 and 500, but using data from Africa, these sites were not above our threshold of 50 % probability of γ > 0. While there were many codons identified to be under significant positive selection in *c(3)G* (16 using African populations, 36 using North American populations), codons under positive selection appeared dispersed along the length of the coding sequence. *cona* showed no particular codons under selection in both *D. melanogaster* samples despite having the highest calculated global ω. This coincides with the failure to detect deviation from neutrality in the polarized MK test (Table 2) and a drastic reduction of ω in *D. melanogaster* according to GA Branch (Fig. 3a).

**Fig. 5:**
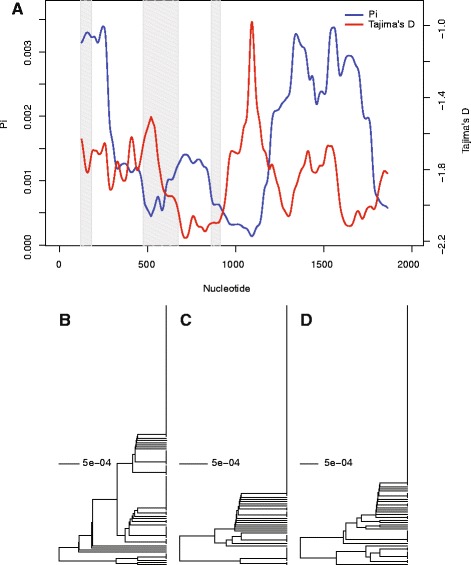
Sliding window estimates of pairwise diversity and Tajima’s D reveal potential signature of positive selection resulting in loss of haplotype diversity. (**a**) Pairwise diversity (π) and Tajima’s D measured in 250 bp windows along the length of *corolla* within the DPGP sequences. Introns are indicated in gray bars. (**b**–**d**) Dendrograms constructed using a HKY model of UPGMA between nucleotides 1-700 (**b**), 701-1300 (**c**), and 1301-1938 (**d**) downstream of the translation start site

Finally, pairwise nucleotide polymorphism (π) was calculated for each SC gene. Overall, there is a similar level of nucleotide diversity in every SC component when compared to π genome-wide and mean π for meiosis genes reported in Anderson, et al. 2009 [42]. The one exception was for *corolla* (Additional file 1: Table S8). *corolla* estimates of synonymous π are considerably lower in both North America and Africa. Considering Tajima’s D, only *corolla* demonstrated a strong negative value (N. Carolina *D* = −2.055, Africa *D* = −2.443, Additional file 1: Table S8), possibly an indication of ongoing positive selection within *corolla.* A sliding window analysis of π and Tajima’s D reveal that the central region of *corolla*, 1000 to 1200 nucleotides downstream of the start codon, is almost entirely lacking polymorphism save one doubleton in the African populations (Fig. 5a) and two singletons within North Carolina (Additional file 1: Figure S5A). In North Carolina populations, 250 bp sliding windows within this region reveal gene regions where *π* = 0 (Additional file 1: Figure S5). Flanking this central region, polymorphism increases and Tajima’s D is negative as many of the site-wise differences can be attributed to singletons, doubletons, and tripletons. Haplotype structure within *corolla* is illustrated with dendrograms constructed using UPGMA [71]. A region of possible recurrent selection shows a higher proportion of individuals carrying a single haplotype with no diversity (Fig. 5c). Crucially, within this span, there are 178 base pairs that are completely monomorphic in both Africa and North Carolina. Flanking this region, there is an increase of diversity and fewer individuals carry the haplotype with no diversity (Fig. 5b, d). This pattern was also observed in the North Carolina population (Additional file 1: Figure S5B–D). Strikingly, within the 178 bp monomorphic span, there are eight non-synonymous substitutions and one synonymous substitution between *D. melanogaster* and *D. simulans* with ω estimated to be 3.40. This also corresponds to the region identified with GammaMap with the highest density of codons characterized by the highest probability that γ > 0 (Fig. 4). This suggests that ongoing positive selection has driven rapid and recurrent change in the protein coding sequence of *corolla*. The low levels of nucleotide diversity within *corolla* in *D. melanogaster* can not be attributed to strong purifying selection since *K*_*a*_*/K*_*s*_ values, another indicator of selective pressure, between *D. melanogaster* and *D. simulans* are high (Additional file 1: Figure S6). In the African populations, the genomic region including *corolla* has reduced polymorphism compared to flanking regions (Additional file 1: Figure S7A). However, the signature is less clear within the North Carolina population (Additional file 1: Figure S7B) possibly due to overall less nucleotide diversity in the DGRP sequences in comparison to the DGPG sequences. This pattern of reduced polymorphism in a 3 kb region is weaker than other signatures of recent positive selection in *D. melanogaster* [81–84]. This may indicate that this pattern of reduced polymorphism in *corolla* may be a remnant of positive selection that is not as recent or as strong as other examples of recent positive selection.

## Discussion

The SC has been identified across diverse eukaryotes with only a few rare exceptions [2, 9, 10, 85]. Homologous protein components of the SC can be found in metazoans ranging from mammals to hydra, indicating that the SC is very likely present at the origin of animals. However, these metazoan SC components are very difficult to detect in Ecdysozoa, including *D. melanogaster* and *C. elegans*, despite the fact that EM studies identify the SC to be structurally similar. Two hypotheses exist for the presence of the SC in the Ecdysozoans: either there has been non-homologous replacement of the SC or an extreme amount of divergence in SC homologs from other lineages.

In support of the hypothesis that a high rate of divergence explains lack of apparent SC protein homology between Ecdysozoa and other metazoans, we presented evidence that the SC is evolving very rapidly within the *Drosophila* genus. Importantly, there is a relationship between the estimated global ω estimates for each protein and the ability to identify orthologs in divergent taxa. Only two genes, *ord* and *c(2)M*, were identified outside of the *Drosophila* genus. These both comprise the lateral element, interact with chromatin, and their ω estimates are the lowest. In contrast, *c(3)G*, *corolla*, and *cona* have higher ω estimates and ortholog identification was more difficult in divergent taxa. Therefore, it is reasonable to conclude that the failure to identify orthologs for SC components outside of the *Drosophila* genus is due to their fast rate of evolution, not necessarily by *de novo* origination within *Drosophila* [18]. Such rapid sequence divergence between orthologs may also suggest that sequence identity is not essential for structural integrity of the SC, despite many *Drosophila*-specific SC components sharing remarkable functional homology with SC components in other eukaryotes. Further resolution of this question may require additional approaches to orthology detection that incorporate structural information and ancestral state reconstruction. Alternatively, proteomic analysis of the SC in species outside the *Drosophila* genus may also identify orthologs that this analysis did not.

We further demonstrate that rapid divergence of sequence identity is not effectively neutral and can in part be explained by prevalent and recurrent positive selection within the *Drosophila* species examined. Using GA Branch, we find that SC evolution is not uniform as originally hypothesized. We provide evidence for a range of ω estimates that have significantly fluctuated across time. GA Branch analysis indicated that *cona*, a component of the CE, had the greatest number of branches with evidence of positive selection. Across the full phylogeny and also the *melanogaster* group, a comparison of M7 and M8 models in PAML identified the strongest signatures of positive selection in *corolla*, also a component of the CE (Table 1); this same gene also posed a challenge for ortholog detection outside of the genus. In contrast, *ord*, a component of the LE, was estimated to have the lowest global ω across the genus and a strong signature of positive selection was observed only when examining the six species within the *melanogaster* group. We found an increased ω for SC components that do not directly interact with chromatin: components of the CE have the highest ω estimates, components of the LE have the lowest and *c(3)G*, which comprises the transverse filament, has an intermediate estimate. A higher rate of evolution for CE proteins in *Drosophila* is concordant with the observation that CE components are more dynamic across metazoans compared to other components [18]. From a structural perspective, the chromatin interaction required of the LE may constrain the rate of evolution. However, CE proteins likely interact with a variety of other meiotic proteins. Therefore, a higher rate of evolution in CE proteins may be partly driven by changes in these interactions.

As the SC is so conserved across eukaryotes, what can explain recurrent positive selection of the SC in *Drosophila*? As previously mentioned, SC components are highly divergent in both *Drosophila* and *Caenorhabditis*. Since both of these genera are in the Ecdysozoa, there may be a shared cause of rapid SC divergence within these two lineages. One shared cause may be the fact that both *D. melanogaster* and *C. elegans* have DSB-independent synapsis. This may lead to reduced constraint on SC components, though it is hard to see how this would lead to recurrent positive selection.

Alternatively, there may be different underlying causes for rapid divergence in these two lineages. There are several features of meiosis that make these lineages unique. *Caenorhabditis* species have holocentric chromosomes with complete crossover interference. *Drosophila* males lack both the SC and meiotic recombination. Thus, multiple forces may independently contribute to the high rate of SC protein evolution in these two lineages.

One possibility is that the rapid evolution and positive selection in SC proteins of *Drosophila* is driven by an interaction between the sex-specific nature of the SC and the rapid turnover of centromeric sequences caused by recurrent bouts of meiotic drive. Previous studies have suggested that sex-specific function can relax selective constraint on a gene and allow it to diverge more freely. This has been proposed to explain the higher divergence of maternally expressed genes such as *bicoid* [86–88]. All of the SC proteins studied have no phenotypic effect in males when mutant, with the exception of ORD which also plays a role in sister chromatid cohesion in the first and second division of meiosis in both sexes [20, 22, 24, 25]. This additional burden of constraint required by being functional in both sexes may explain why *ord* has the lowest ω value among the SC genes examined.

Because the SC is expressed only in females, it may be particularly influenced by rapid evolution of centromeric sequences driven by meiotic drive. In contrast to male meiosis where all four meiotic products become functional gametes, only one of four meiotic products becomes the egg pronucleus, with the remaining three forming polar bodies. Therefore, strong selection in female meiosis can favor a centromere that is biased to enter the pronucleus over an opposing centromere. A centromeric variant that strongly distorts meiosis in its favor will sweep through the population even though it may convey deleterious effects such as interfering in male spermatogenesis [89–91]. This form of competition has been proposed drive rapid evolution of centromeric sequences [92–97]. Rapid evolution of centromeric sequences arising from centromere drive has also been proposed to explain signatures of positive selection on centromere-associated proteins such as the centromeric variant of histone H3 [96, 98, 99].

SC components also have specialized functions at centromeres. Across diverse organisms, early centromeric associations are mediated by components of the SC [100]. For example, in budding yeast, the TF protein Zip1 is required for early centromere coupling [101], though not through formation of the SC per se [102]. In *Drosophila*, SC components have the unique property of mediating centromere pairing in mitotically dividing germ cells [103, 104]. Additionally, the *Drosophila* SC is essential for centromere synapsis within the chromocenter [105, 106] where the SC is first assembled prior to assembling along the length of the chromosome arms. Finally, across diverse organisms, the SC persists in centromeric regions long after SC disassembly from the euchromatin [100]. This persistence likely facilitates proper chromosomal segregation [102].

Due to these multiple functions at the centromere, and as has been proposed for centromeric histones [43, 93], positive selection in SC components may be driven by the need to accommodate rapid turnover of centromere sequences driven by bouts of centromere drive in female meiosis. This signal may be enhanced by the sex-specific nature of the SC in *Drosophila*. Additional support for this hypothesis lies in the conservation of *c(2)M* when compared to other SC components. Our analyses showed few signs of positive selection in *c(2)M* beyond its high global ω, which was higher than *ord*. In the studies of SC centromere association, *c(2)M* mutants either showed partially reduced centromere clustering [105] or no effect [106]. *c(2)M* may show a weaker signature of positive selection compared to other SC components because it has a limited role in centromeric clustering.

It is also worth noting that the SC plays a crucial role in establishing the landscape of recombination in meiosis. Recent studies have shown that selection may act to modify recombination landscapes as a means to reduce the cost of female meiotic drive, particularly by modulating recombination rates near centromeres [107]. Previous studies have also shown that the centromere can vary significantly in its effects on local recombination in closely related species of the *D. melanogaster* group [108]. Overall, we propose that positive selection may jointly arise from the role that SC components have at rapidly evolving centromeres and modulation of recombination rates in these regions. A combination of these forces, along with sex-specificity, may play an important role in driving rapid evolution of this highly conserved structure in *Drosophila.*

## Conclusions

The SC shows little sequence conservation across eukaryotes despite its conserved function in meiotic segregation and recombination. The genes comprising the *Drosophila* SC show almost no apparent homology when compared to SC components in other model organisms. We have determined that the SC components in *Drosophila* are evolving rapidly and their ω estimates are higher than observed for most genes. We conclude that this can be partly explained by positive selection detected in nearly every SC gene. This contrasts to our understanding of the SC as a conserved structure necessary for fertility. We propose that the combination of the female-exclusive function of the SC within *Drosophila*, its role in meiotic recombination, and its interaction with centromeres is driving the rapid evolution of the SC within *Drosophila*.

## Availability of data and material

The datasets supporting the conclusions of this article are included within this article and its Additional files 2, 3 and 4.

## Additional files

Additional file 1: Supplementary Tables and Figures. Tables summarizing orthology search and detection, likelihood values for PAML tests, and population genetic parameters estimated from the data. Figures describing ω calculated from HyPhy, GA Branch using MUSCLE- and PRANK-aligned sequences, the GammaMap output for the DGRP, and per-site differences and Tajima’s D estimates along the length of *corolla* for the DPGP. (PDF 1109 kb) Additional file 2: Aligned sequences of *Drosophila* SC genes. Text file containing the aligned sequences of each SC gene from the respective *Drosophila* species. Alignments were performed by MAFFT, MUSCLE, and PRANK. (TXT 1116 kb) Additional file 3: Aligned *D. melanogaster* population sequences from the *Drosophila* Population Genomics Project (DPGP). Sequences were aligned to *D. simulans* and *D. yakuba*. Sequences that did not align or were missing data were removed prior to the MK tests. (TXT 676 kb) Additional file 4: Aligned *D. melanogaster* population sequences from the *Drosophila* Genetics Reference Panel (DGRP). Sequences were aligned to *D. simulans* and *D. yakuba*. Sequences that did not align or were missing data were removed prior to the MK tests. (TXT 929 kb)

## Abbreviations

CE: central element
DFE: distribution of fitness effects
DGRP: Drosophila Genomics Reference Panel
DPGP: Drosophila Population Genomics Project
LE: lateral element
MK tests: McDonald-Kreitman tests
SC: synaptonemal complex
TF: transverse filament
