# Supplementary material for: Holding it together: rapid evolution and positive selection in the synaptonemal complex of Drosophila
Source: BMC Evol Biol. 2016 May 5;16:91. doi: 10.1186/s12862-016-0670-8 (PMC4857336; doi:10.1186/s12862-016-0670-8)
Supplement: Additional file 1: — Supplementary Tables and Figures. Tables summarizing orthology search and detection, likelihood values for PAML tests, and population genetic parameters estimated from the data. Figures describing ω calculated from HyPhy, GA Branch using MUSCLE- and PRANK-aligned sequences, the GammaMap output for the DGRP, and per-site differences and Tajima’s D estimates along the length of corolla for the DPGP. (PDF 1109 kb) [file 12862_2016_670_MOESM1_ESM.pdf]

Holding it together: Rapid evolution and positive selection in the synaptonemal complex of *Drosophila*

Supplemental Figures and Tables

Lucas W. Hemmer[1*] and Justin P. Blumenstiel[1]

[1]Department of Ecology and Evolutionary Biology, University of Kansas, Lawrence Kansas 66045

*Corresponding Author:

Emails:

LWH: lhemmer@ku.edu

JPB: jblumens@ku.edu

Figure S1: The global ω of each SC gene calculated in HyPhy using a GTR nucleotide substitution model with 95% confidence intervals. The ratio remains relatively consistent for each alignment program used (MAFFT, MUSCLE, and PRANK) and divergence times. It is also consistent with the PAML-derived results albeit slightly higher ω estimates.

Figure S2: MUSCLE-aligned GA Branch diagrams of A) Ord, B) C(2)M, C) C(3)G, D) Corolla, and E) Cona. Branch colors correspond with the associated ω ratio and posterior probabilities of positive selection are listed as a percent on each branch.

Figure S3: PRANK-aligned GA Branch diagrams of A) Ord, B) C(2)M, C) C(3)G, D) Corolla, and E) Cona. Branch colors correspond with the associated ω ratio and posterior probabilities of positive selection are listed as a percent on each branch.

Figure S4: GammaMap figures of the DGRP (North Carolina) *D. melanogaster* population sequences. In concordance with Wilson *et al.* 2011, a codon is under significant signature of selection when the posterior probabilities of selection (lines) are greater than 0.5. Vertical bars illustrate polymorphisms in D. melanogaster and the substitutions are the circular dots. The colors correspond to *D. melanogaster* non-synonymous (red) and synonymous (dark green) variants as well as *D. simulans* non-synonymous (orange) and synonymous (light green) variants. Estimated number of selected codons is indicated in the upper right of each plot. Additionally, a red bar indicates the region of the hypothesized selective sweep in *corolla*.

Figure S5: Sliding window estimates of pairwise divergence and Tajima's D reveal recent positive selection resulting in loss of haplotype diversity. (A) Pairwise differences (π) and Tajima's D measured in 250 bp windows along the length of *corolla* within the DGRP sequences. Introns are indicated in gray bars. Black lines indicate portions of the gene used in the dendrograms for parts B, C, and D. (B-D) Dendrograms constructed using a HKY model of UPGMA between nucleotides 1-700 (B), 701-1300 (C), and 1301-1938 (D) downstream of the translation start site.

Figure S6: Sliding window estimates of pairwise divergence and K_a/K_s show little correlation in selection between *D. melanogaster* and *D. simulans*. Pairwise differences (π) and divergence (K_a/K_s) measured in 250 bp windows along the length of *corolla* in the DGPG sequences (A) and the DGRP sequences (B). Introns are indicated in gray bars.

Figure S7: Sliding window estimates of pairwise divergence and Tajima's D reveal reduced polymorphism surrounding *corolla* in Africa but not North Carolina. Pairwise differences (π) and Tajima's D measured in 800 bp windows along the genomic region containing *corolla* and 5 kb upstream and downstream of the gene in the DGPG sequences (A) and DGRP sequences (B). Introns are indicated in gray bars and the length of the *corolla* gene from the starting codon to the stop codon is indicated by the black bar.

Figure S1

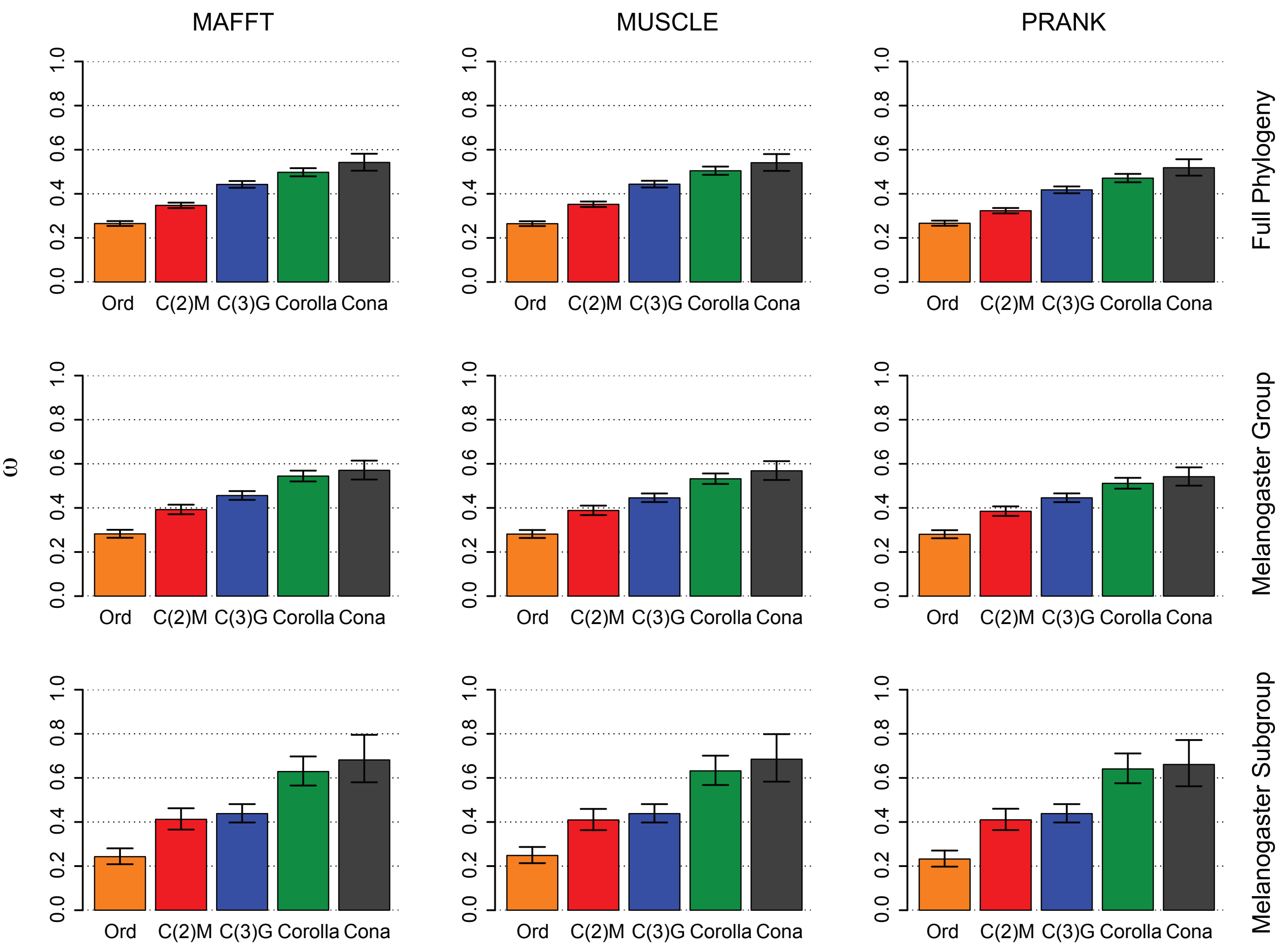
MAFFT
MUSCLE
PRANK
Full Phylogeny
Melanogaster Group
Melanogaster Subgroup
ω
0.0
0.2
0.4
0.6
0.8
1.0
Ord
C(2)M
C(3)G
Corolla
Cona

Figure S2

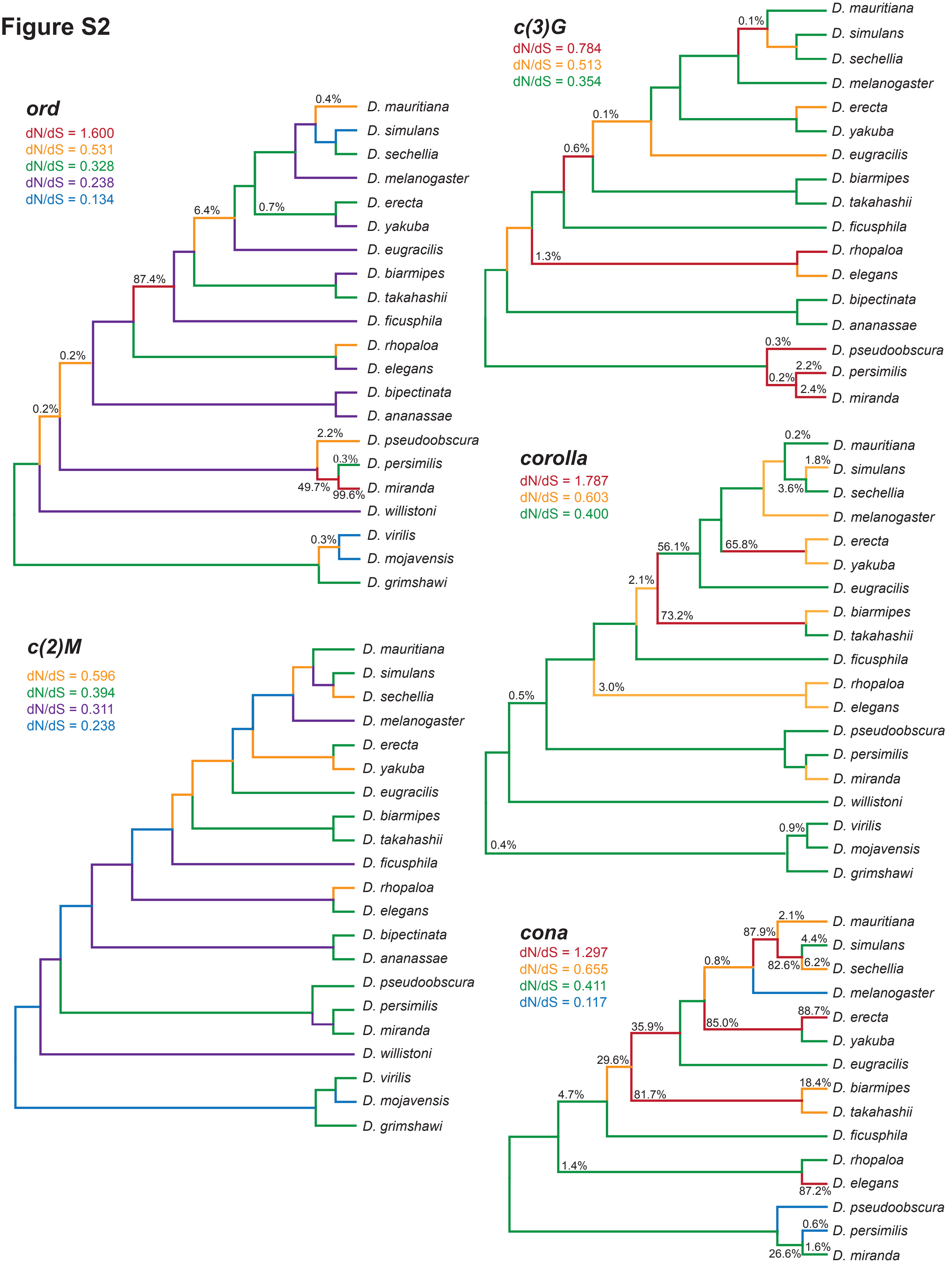
ord
dN/dS = 1.600
dN/dS = 0.531
dN/dS = 0.328
dN/dS = 0.238
dN/dS = 0.134
0.4%
6.4%
0.7%
87.4%
0.2%
0.2%
2.2%
0.3%
49.7%
99.6%
0.3%
D. mauritiana
D. simulans
D. sechellia
D. melanogaster
D. erecta
D. yakuba
D. eugracilis
D. biarmipes
D. takahashii
D. ficusphila
D. rhopaloa
D. elegans
D. bipectinata
D. ananassae
D. pseudoobscura
D. persimilis
D. miranda
D. willistoni
D. virilis
D. mojavensis
D. grimshawi
c(2)M
dN/dS = 0.596
dN/dS = 0.394
dN/dS = 0.311
dN/dS = 0.238
D. mauritiana
D. simulans
D. sechellia
D. melanogaster
D. erecta
D. yakuba
D. eugracilis
D. biarmipes
D. takahashii
D. ficusphila
D. rhopaloa
D. elegans
D. bipectinata
D. ananassae
D. pseudoobscura
D. persimilis
D. miranda
D. willistoni
D. virilis
D. mojavensis
D. grimshawi
c(3)G
dN/dS = 0.784
dN/dS = 0.513
dN/dS = 0.354
0.1%
0.1%
0.6%
1.3%
0.3%
2.2%
0.2%
2.4%
D. mauritiana
D. simulans
D. sechellia
D. melanogaster
D. erecta
D. yakuba
D. eugracilis
D. biarmipes
D. takahashii
D. ficusphila
D. rhopaloa
D. elegans
D. bipectinata
D. ananassae
D. pseudoobscura
D. persimilis
D. miranda
corolla
dN/dS = 1.787
dN/dS = 0.603
dN/dS = 0.400
0.2%
1.8%
3.6%
56.1%
65.8%
2.1%
73.2%
0.5%
3.0%
0.9%
0.4%
D. mauritiana
D. simulans
D. sechellia
D. melanogaster
D. erecta
D. yakuba
D. eugracilis
D. biarmipes
D. takahashii
D. ficusphila
D. rhopaloa
D. elegans
D. pseudoobscura
D. persimilis
D. miranda
D. willistoni
D. virilis
D. mojavensis
D. grimshawi
cona
dN/dS = 1.297
dN/dS = 0.655
dN/dS = 0.411
dN/dS = 0.117
2.1%
87.9%
4.4%
0.8%
82.6%
6.2%
88.7%
35.9%
85.0%
29.6%
18.4%
4.7%
81.7%
1.4%
87.2%
0.6%
26.6%
1.6%
D. mauritiana
D. simulans
D. sechellia
D. melanogaster
D. erecta
D. yakuba
D. eugracilis
D. biarmipes
D. takahashii
D. ficusphila
D. rhopaloa
D. elegans
D. pseudoobscura
D. persimilis
D. miranda

Figure S3

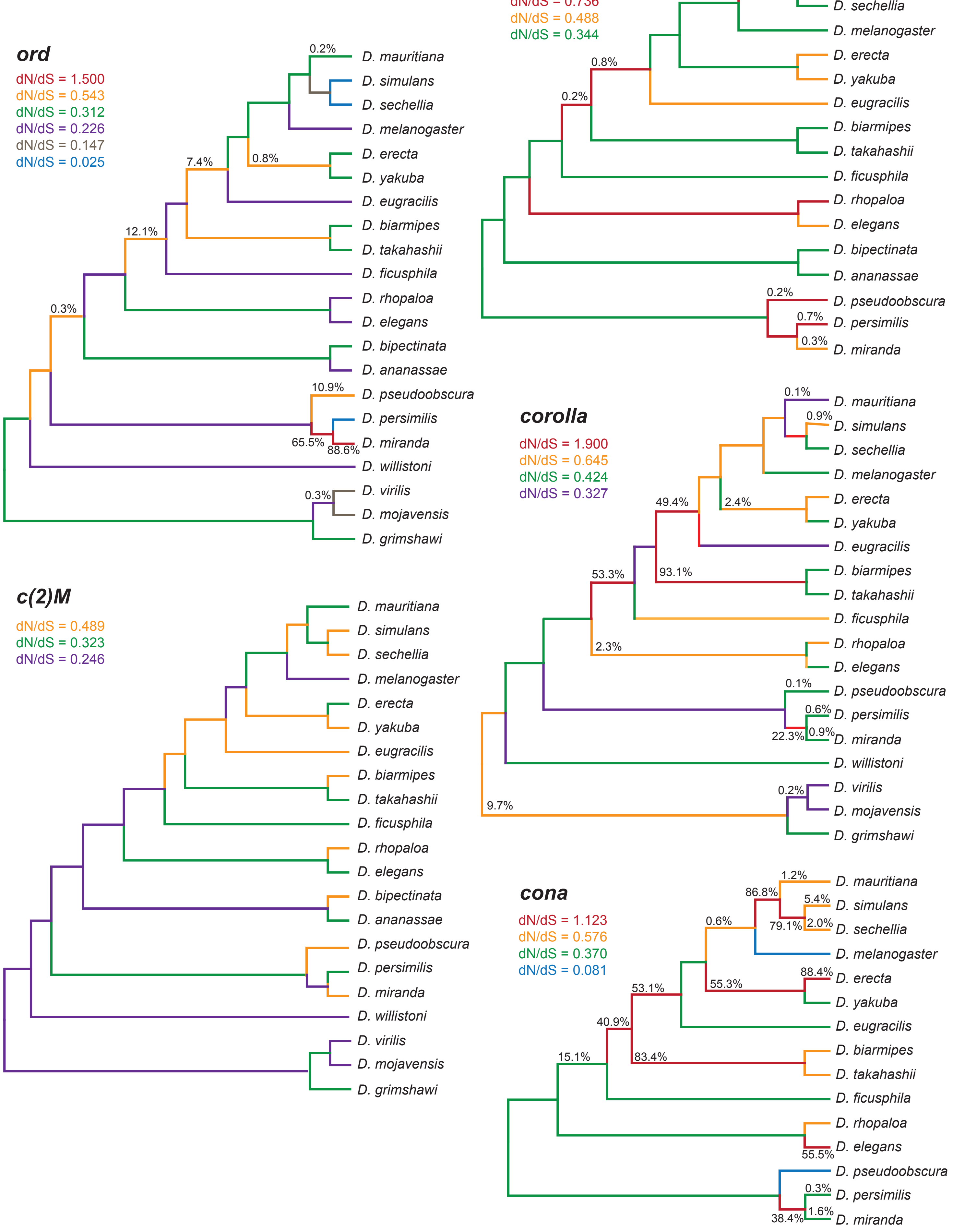
ord
dN/dS = 1.500
dN/dS = 0.543
dN/dS = 0.312
dN/dS = 0.226
dN/dS = 0.147
dN/dS = 0.025
0.2%
7.4%
0.8%
12.1%
0.3%
10.9%
65.5%
88.6%
0.3%
D. mauritiana
D. simulans
D. sechellia
D. melanogaster
D. erecta
D. yakuba
D. eugracilis
D. biarmipes
D. takahashii
D. ficusphila
D. rhopaloa
D. elegans
D. bipectinata
D. ananassae
D. pseudoobscura
D. persimilis
D. miranda
D. willistoni
D. virilis
D. mojavensis
D. grimshawi
c(3)G
dN/dS = 0.736
dN/dS = 0.488
dN/dS = 0.344
0.8%
0.2%
0.2%
0.7%
0.3%
D. mauritiana
D. simulans
D. sechellia
D. melanogaster
D. erecta
D. yakuba
D. eugracilis
D. biarmipes
D. takahashii
D. ficusphila
D. rhopaloa
D. elegans
D. bipectinata
D. ananassae
D. pseudoobscura
D. persimilis
D. miranda
c(2)M
dN/dS = 0.489
dN/dS = 0.323
dN/dS = 0.246
D. mauritiana
D. simulans
D. sechellia
D. melanogaster
D. erecta
D. yakuba
D. eugracilis
D. biarmipes
D. takahashii
D. ficusphila
D. rhopaloa
D. elegans
D. bipectinata
D. ananassae
D. pseudoobscura
D. persimilis
D. miranda
D. willistoni
D. virilis
D. mojavensis
D. grimshawi
corolla
dN/dS = 1.900
dN/dS = 0.645
dN/dS = 0.424
dN/dS = 0.327
0.1%
0.9%
49.4%
2.4%
53.3%
93.1%
2.3%
0.1%
0.6%
22.3%
0.9%
0.2%
9.7%
D. mauritiana
D. simulans
D. sechellia
D. melanogaster
D. erecta
D. yakuba
D. eugracilis
D. biarmipes
D. takahashii
D. ficusphila
D. rhopaloa
D. elegans
D. pseudoobscura
D. persimilis
D. miranda
D. willistoni
D. virilis
D. mojavensis
D. grimshawi
cona
dN/dS = 1.123
dN/dS = 0.576
dN/dS = 0.370
dN/dS = 0.081
1.2%
86.8%
5.4%
0.6%
79.1%
2.0%
88.4%
53.1%
55.3%
40.9%
15.1%
83.4%
55.5%
0.3%
38.4%
1.6%
D. mauritiana
D. simulans
D. sechellia
D. melanogaster
D. erecta
D. yakuba
D. eugracilis
D. biarmipes
D. takahashii
D. ficusphila
D. rhopaloa
D. elegans
D. pseudoobscura
D. persimilis
D. miranda

Figure S4

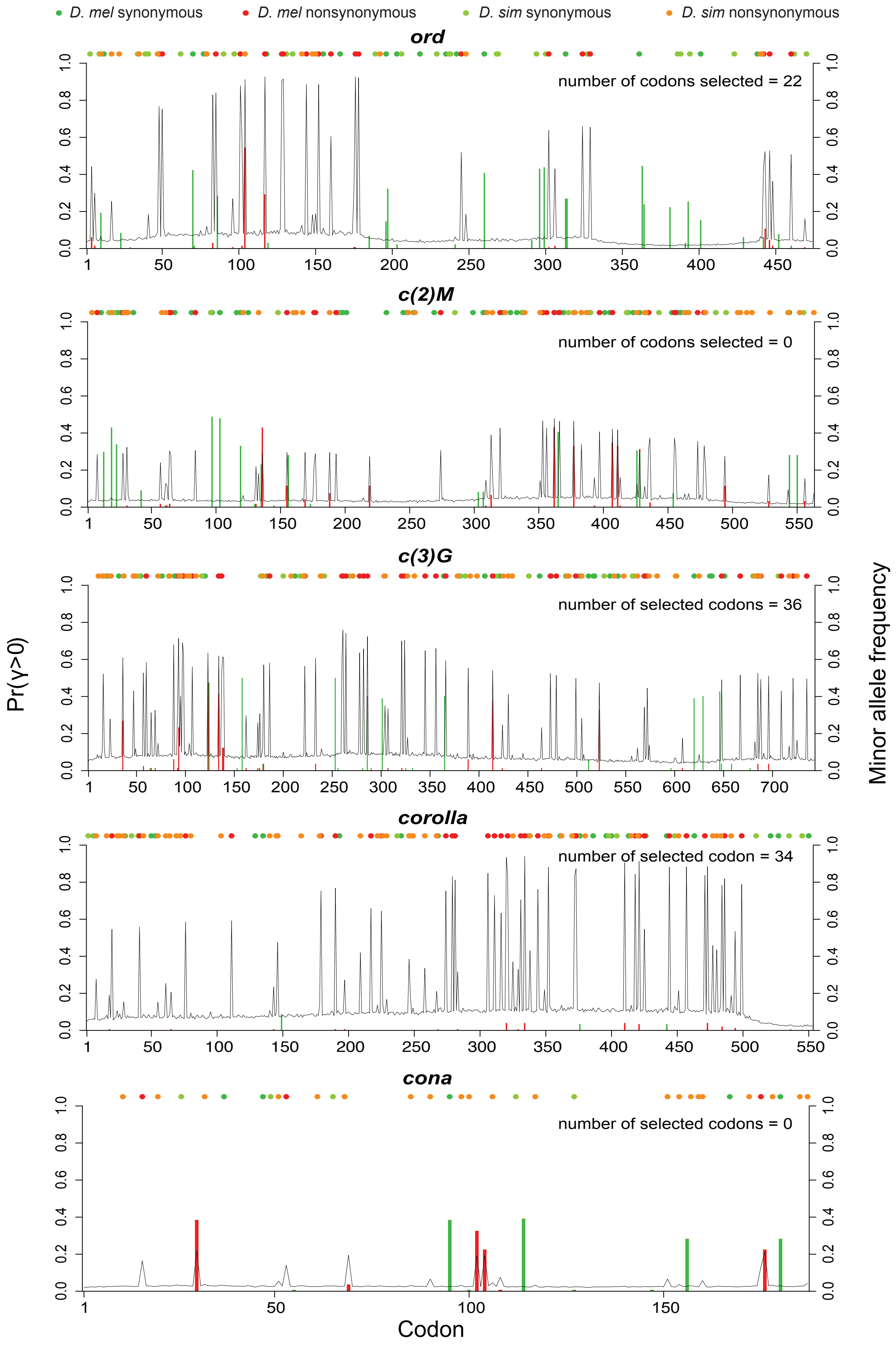
D. mel synonymous
D. mel nonsynonymous
D. sim synonymous
D. sim nonsynonymous
ord
number of codons selected = 22
c(2)M
number of codons selected = 0
c(3)G
number of selected codons = 36
corolla
number of selected codon = 34
cona
number of selected codons = 0
Pr(γ>0)
Minor allele frequency
Codon

Figure S5

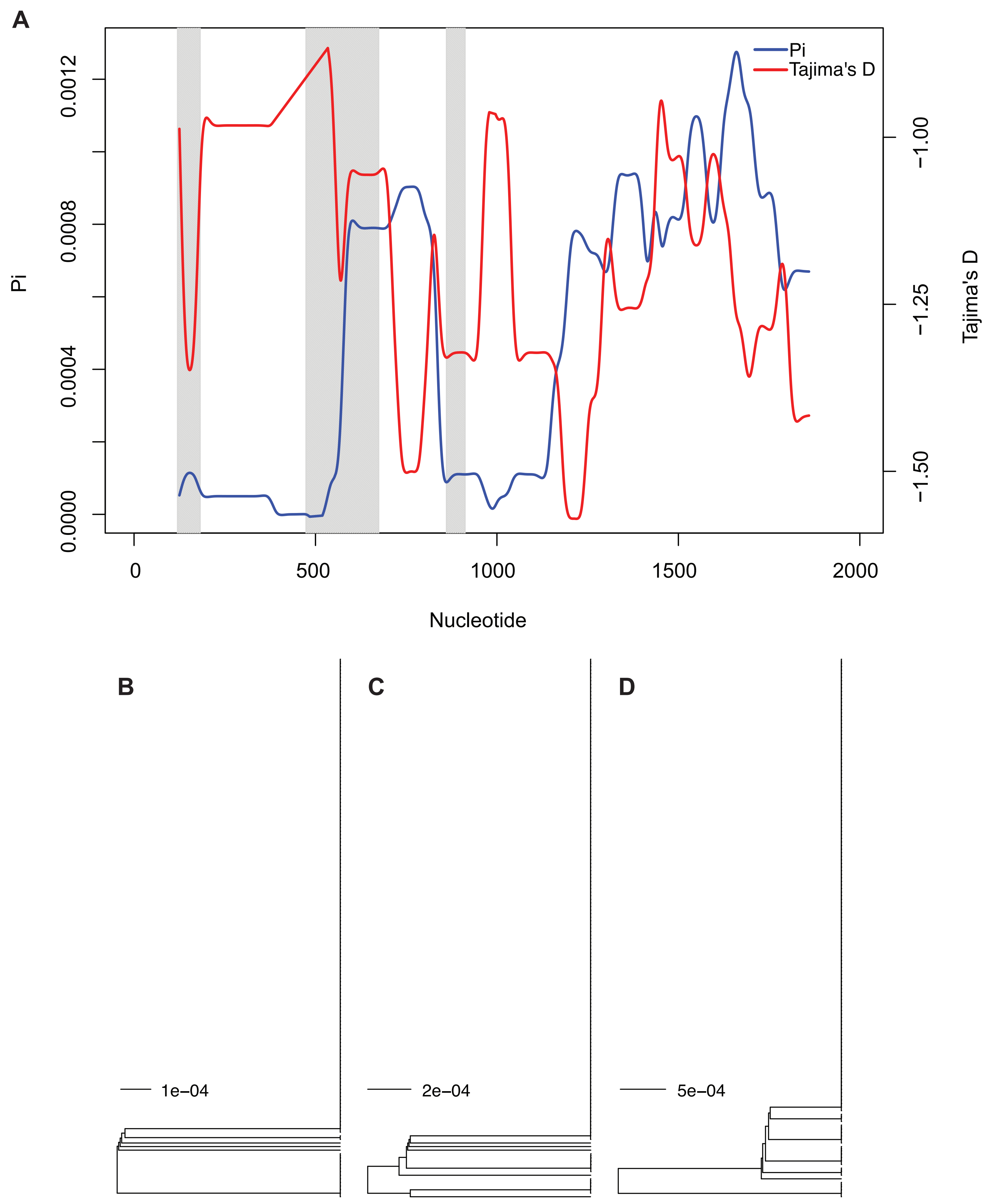
A
Pi
Tajima's D
0.0000
0.0004
0.0008
0.0012
−1.00
−1.25
−1.50
0
500
1000
1500
2000
Nucleotide
B
C
D
1e−04
2e−04
5e−04

Figure S6

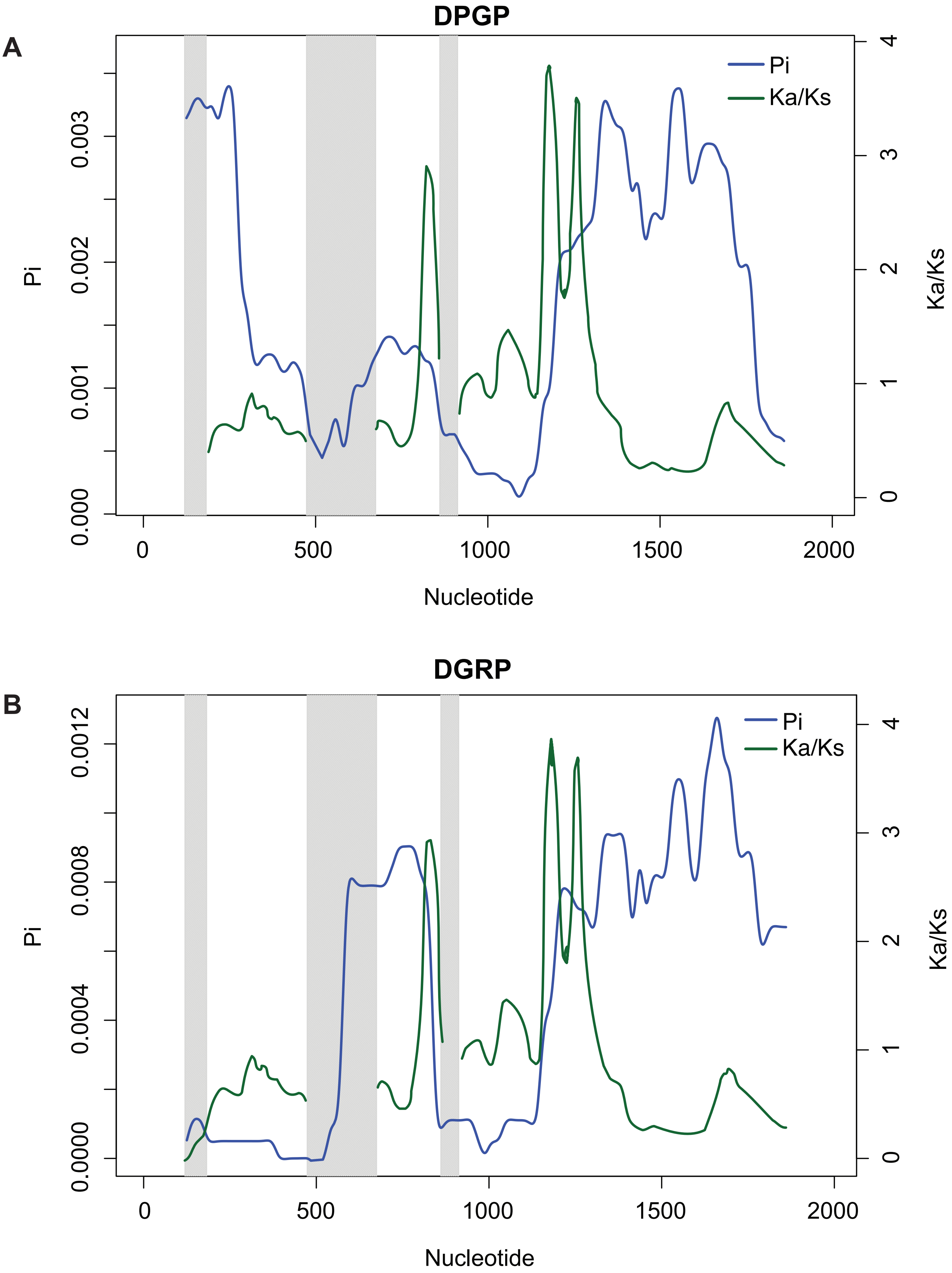
A
DPGP
Pi
Ka/Ks
0.000
0.001
0.002
0.003
0
1
2
3
4
0
500
1000
1500
2000
Nucleotide
B
DGRP
Pi
Ka/Ks
0.0000
0.0004
0.0008
0.0012
0
1
2
3
4
0
500
1000
1500
2000
Nucleotide

Figure S7

A

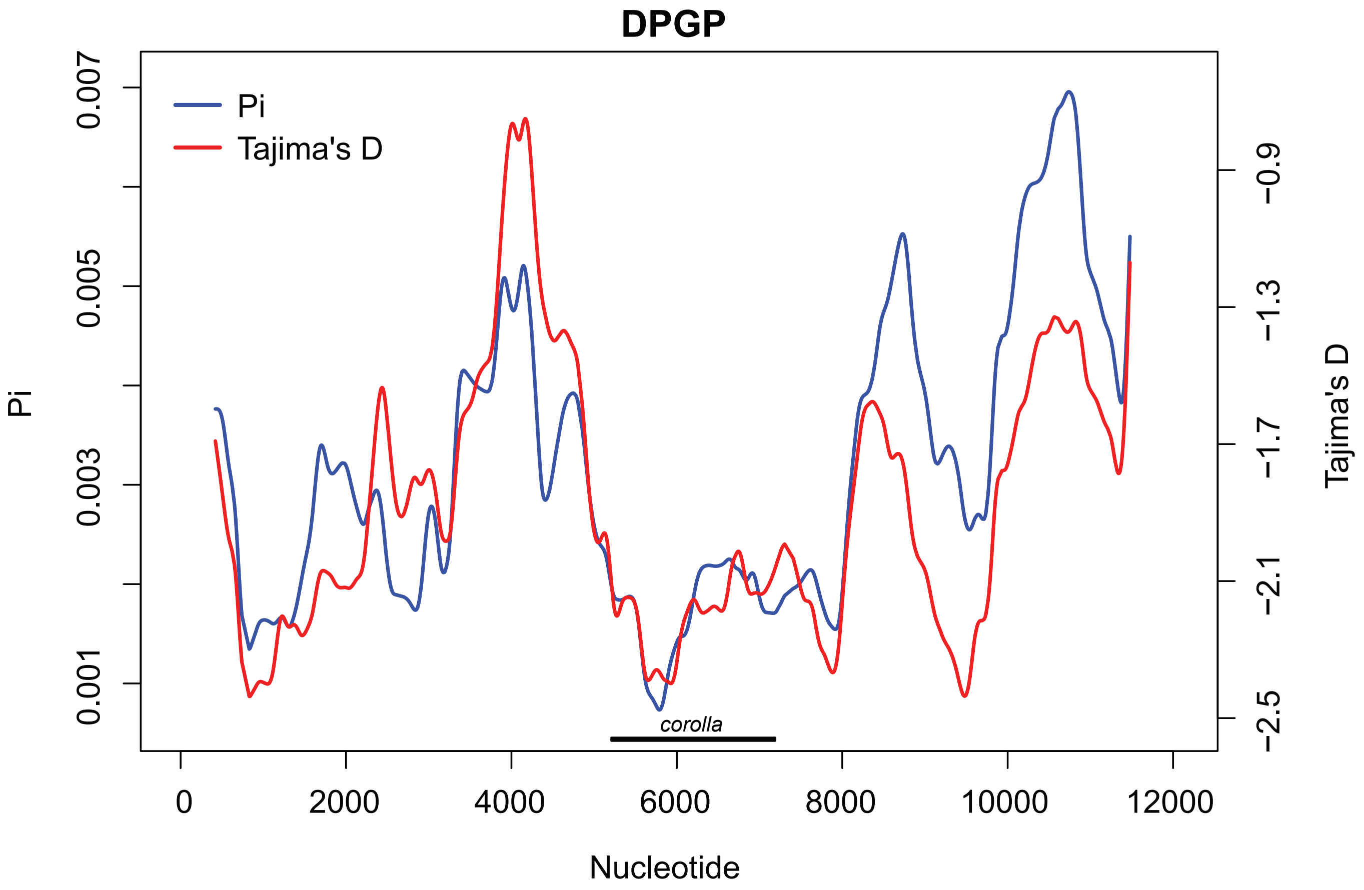
DPGP
Pi
Tajima's D
0.007
0.005
0.003
0.001
-0.9
-1.3
-1.7
-2.1
-2.5
corolla
0
2000
4000
6000
8000
10000
12000
Nucleotide
Pi
Tajima's D

B

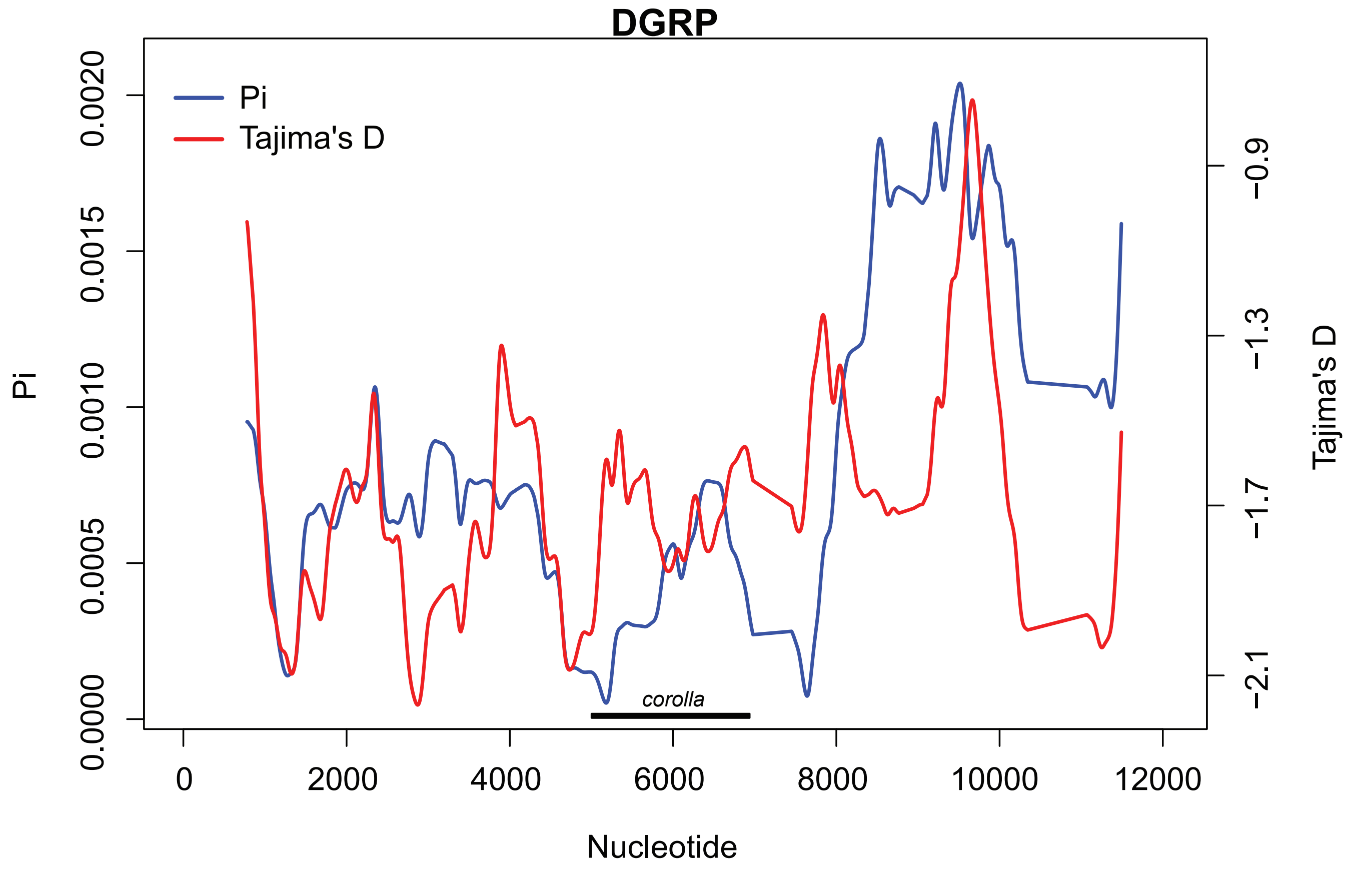
DGRP
Pi
Tajima's D
0.0020
0.0015
0.0010
0.0005
0.0000
-0.9
-1.3
-1.7
-2.1
corolla
0
2000
4000
6000
8000
10000
12000
Nucleotide
Pi
Tajima's D

Supplmental Tables

Table S1: Syntenic checks for all confirmed orthologs. "X" marks the presence of one of the four nearby genes (2 on the Left and 2 on the Right). "Y" marks certainty of synteny while "N" is not syntenic. A "?" marks an ambiguous identified ortholog, either due to the absence of nearby syntenic genes or being located on the wrong Muller Element. Some ambiguous genes were included anyway if they are double marked with a "Y?" or "N?".

Gene	Species	Contig/Chromosome	2L Gene	1L Gene	1R Gene	2R Gene	Muller Element	Syntenic
			CG12782	*CG13540*	*CG3124*	*CG13541*		
	D. ananassae	scaffold_13266	X	-	X	-	C	Y
	D. erecta	scaffold_4845	X	X	X	X	BC	Y
	D. grimshawi	scaffold_15112	-	-	X	-	C	Y
	D. melanogaster	2R	X	X	X	X	C	Y
	D. mojavensis	scaffold_6496	-	-	X	-	C	Y
Ord	*D. persimilis*	scaffold_2	-	-	X	-	C	Y
	D. pseudoobscura	3	-	-	X	-	C	Y
	D. sechilia	scaffold_9	X	X	X	X	C	Y
	D. simulans	2R	X	X	X	X	C	Y
	D. virilis	scaffold_12875	-	-	X	-	C	Y
	D. willistoni	scf2_1100000004512	-	-	-	-	C	?
	D. yakuba	2R	X	X	X	X	BC	Y
			CG17328	*CG5869*	*CG5861*	*Syx5*		
C(2)M	*D. ananassae*	scaffold_12916	-	-	-	-	B	Y?

	D. erecta	scaffold_4929	X	X	X	X	BC	Y
	D. grimshawi	scaffold_15252	X	X	X	X	B	Y
	D. melanogaster	2L	X	X	X	X	B	Y
	D. mojavensis	scaffold_6500	X	X	X	X	B	Y
	D. persimilis	scaffold_8	X	X	X	X	B	Y
	D. pseudoobscura	4_group2	X	X	X	X	B	Y
	D. sechilia	scaffold_5	X	X	X	X	B	Y
		scaffold_6278	-	-	-	-	?	N
	D. simulans	2L	X	X	X	X	B	Y
	D. virilis	scaffold_12963	X	X	X	X	B	Y
		scaffold_12970	-	-	-	-	A	N
	D. willistoni	scf_1100000004516	-	-	X	X	B	Y
	D. yakuba	2L	X	X	X	X	BC	Y
			CG9590	*CG9589*	*Acyp2*	*wah*		
	D. ananassae	scaffold_13266	-	-	-	-	C	?
	D. erecta	scaffold_4770	X	X	X	X	E	Y
		scaffold_4770	X	X	X	X	E	Y
	D. grimshawi	-	-	-	-	-	-	-
	D. melanogaster	3R	X	X	X	X	E	Y
C(3)G	*D. mojavensis*	-	-	-	-	-	-	-
	D. persimilis	scaffold_0	-	-	-	-	E	?
	D. pseudoobscura	2	-	-	-	-	E	?
	D. sechilia	scaffold_0	X	X	X	X	E	Y
	D. simulans	3R	X	X		X	E	Y
	D. virilis	-	-	-	-	-	-	-
	D. willistoni	-	-	-	-	-	-	-
	D. yakuba	3R	X	X	X	X	E	Y
Corolla			*stas*	*CG8326*	*CG5703*	*CG8289*		

	D. ananassae		-	-	-	-	-	
	D. erecta	scaffold_4690	X	X	X	X	A	Y
	D. grimshawi	scaffold_15074	-	-	-	-	E	?
	D. melanogaster	X	X	X	X	X	A	Y
	D. mojavensis	X	X	-	-	-	A	Y
	D. persimilis	scaffold_17	X	X	X	X	A	Y
	D. pseudoobscura	XL_group1e	X	X	X	X	A	Y
	D. sechilia	scaffold_17	X	X	X	X	A	Y
	D. simulans	X	X	X	X	X	A	Y
	D. virilis	scaffold_12970	X	-	-	-	A	Y
	D. willistoni	-	-	-	-	-	-	-
	D. yakuba	X	X	X	X	X	A	Y
Cona			*CG7675*	*CG14309*	*Vha100-4*	*Vha100-2*		
	D. ananassae	-	-	-	-	-	-	-
	D. erecta	scaffold_4770	X	X	X	X	E	Y
	D. grimshawi	-	-	-	-	-	-	-
	D. melanogaster	3R	X	X	X	X	E	Y
	D. mojavensis	-	-	-	-	-	-	-
	D. persimilis	scaffold_17	-	-	-	-	A	N
	D. pseudoobscura	XL_group1e	-	-	-	-	A	N
	D. sechilia	scaffold_5	X	X	X	X	E	
	D. simulans	3R	X	X	X	X	E	
	D. virilis	-	-	-	-	-	-	-
	D. willistoni	scf2_1100000004963	-	-	-	-	A	N
	D. yakuba	3R	X	X	X	X	E	Y
		v2_chrUn_2917	-	-	-	-	?	?

Table S2: Orthology searches of all 5 genes amongst PhylomeDB, OrthoDB, and HMMER.

Gene	Specis	PhylomeDB	OrthoDB	HMMER	HMMER E-value
Ord	*D. ananassae*	FBgn0090337	FBgn0090337	FBgn0090337	3.3E-134
	D. erecta	FBgn0112252	FBgn0112252	FBgn0112252	7.6E-270
	D. grimshawi	FBgn0130509	FBgn0130509	FBgn0130509	9.4E-104
	D. melanogaster	FBgn0003009	FBgn0003009	FBgn0003009	0.0E+00
	D. mojavensis	FBgn0143855	FBgn0143855	FBgn0143855	1.5E-116
	D. persimilis	FBgn0148894	FBgn0148894	FBgn0148894	9.6E-127
	D. pseudoobscura	FBgn0076207	FBgn0076207	FBgn0076207	1.4E-126
	D. sechilia	FBgn0170485	FBgn0170485	FBgn0170485	1.3E-236
	D. simulans	FBgn0196381	FBgn0196381	FBgn0196381	1.5E-302
	D. virilis	FBgn0208105	FBgn0208105	FBgn0208105	2.3E-116
	D. willistoni	FBgn0221554	-	FBgn0221554	4.4E-60
	D. yakuba	FBgn0229389	FBgn0229389	FBgn0229389	1.5E-266
C(2)M	*D. ananassae*	FBgn0091606	FBgn0091606	FBgn0091606	1.7E-104
	D. erecta	FBgn0117325	FBgn0117325	FBgn0117325	3.0E-295
	D. grimshawi	FBgn0117794	FBgn0117794	FBgn0117794	2.6E-66
	D. melanogaster	FBgn0028525	FBgn0028525	FBgn0028525	0.0E+00
	D. mojavensis	FBgn0139819	FBgn0139819	FBgn0139819	2.8E-76
	D. persimilis	FBgn0153925	FBgn0153925	FBgn0153925	2.3E-110
	D. pseudoobscura	FBgn0078066	FBgn0078066	FBgn0078066	1.0E-115
	D. sechilia	FBgn0173571	FBgn0173571	FBgn0173571	0.0E+00
	D. simulans	FBgn0195408	FBgn0195408	FBgn0195408	1.3E-272
	D. virilis	FBgn0197897	FBgn0197897	FBgn0197897	2.3E-116
	D. willistoni	-	FBgn0206237	FBgn0206237	4.4E-60
	D. yakuba	FBgn0238700	FBgn0238700	FBgn0238700	1.5E-266
C(3)G	*D. ananassae*	-	-	-	-
	D. erecta	FBgn0112578	FBgn0112578	FBgn0112578	0.0E+00
		FBgn0112568	FBgn0112568	FBgn0112568	5.0E-185
	D. grimshawi	-	-	-	-
	D. melanogaster	FBgn0000246	FBgn0000246	FBgn0000246	0.0E+00
	D. mojavensis	-	-	-	-
	D. persimilis	FBgn0161282	FBgn0161282	FBgn0161282	1.1E-25
	D. pseudoobscura	FBgn0248078	FBgn0248078	FBgn0248078	3.8E-08
	D. sechilia	FBgn0180610	FBgn0180610	FBgn0180610	0.0E+00
	D. simulans	FBgn0191803	FBgn0191803	FBgn0191803	0.0E+00
	D. virilis			FBgn0202545	6.9E-04
	D. willistoni	FBgn0212362	FBgn0212362	FBgn0212362	2.6E-10
	D. yakuba	FBgn0243389	FBgn0243389	FBgn0243389	2.5E-305
Corolla	*D. ananassae*	-	FBgn0099545	FBgn0099545	3.6E-08
	D. erecta	FBgn0110389	FBgn0110389	FBgn0110389	2.8E-200

	D. grimshawi	FBgn0120438	FBgn0120438	FBgn0120438	5.3E-17
	D. melanogaster	FBgn0030852	FBgn0030852	FBgn0030852	0.0E+00
	D. mojavensis	FBgn0137382	FBgn0137382	FBgn0137382	1.8E-13
	D. persimilis	FBgn0157987	FBgn0157987	FBgn0157987	2.6E-17
	D. pseudoobscura	FBgn0250506	FBgn0250506	FBgn0250506	7.0E-16
	D. sechilia	FBgn0168236	FBgn0168236	FBgn0168236	4.8E-291
	D. simulans	FBgn0188911	FBgn0188911	FBgn0188911	3.9E-292
	D. virilis	FBgn0206426	FBgn0206426	FBgn0206426	1.4E-17
	D. willistoni	FBgn0227567	FBgn0227567	FBgn0227567	2.5E-08
	D. yakuba	FBgn0233155	FBgn0233155	FBgn0233155	1.3E-217
Cona	*D. ananassae*	-	FBgn0097212	FBgn0097212	2.9E-03
	D. erecta	FBgn0115037	FBgn0115037	FBgn0115037	9.6E-67
	D. grimshawi	-	-	FBgn0132111	1.9E-03
	D. melanogaster	-	-	-	-
	D. mojavensis	-	-	FBgn0138867	1.8E-02
	D. persimilis	FBgn0157804	FBgn0157804	FBgn0157804	6.4E-10
	D. pseudoobscura	FBgn0248220	FBgn0248220	FBgn0248220	9.5E-11
	D. sechilia	FBgn0172774	FBgn0172774	FBgn0172774	2.1E-104
	D. simulans	FBgn0190734	FBgn0190734	FBgn0190734	7.8E-105
	D. virilis	-	-	FBgn0203581	1.3E-02
	D. willistoni	-	-	-	-
	D. yakuba	FBgn0242600	FBgn0242600	FBgn0242600	2.2E-69
		FBgn0232288	FBgn0232288	FBgn0232288	1.5E-65

Table S3: Ord orthologs confirmed by reciprocal BLAST back to the *D. melanogaster* genome.

Species	Accession Number	Method	Seed	E-value
D. melanogaster[a]	CG3134	tBLASTn		0.00[b]
D. simulans[a]	GD25609	tBLASTn	*D. melanogaster*	0.00[b]
D. mauritiana[a]	NA	tBLASTn	*D. melanogaster*	0.00[b]
D. sechellia[a]	GM15567	tBLASTn	*D. melanogaster*	0.00[b]
D. yakuba[a]	GE11589	tBLASTn	*D. melanogaster*	0.00[b]
D. erecta[a]	GG20053	tBLASTn	*D. melanogaster*	0.00[b]
D. eugracilis[a]	KB465221.1	tBLASTn	*D. melanogaster*	0.00[b]
D. takahashii[a]	KB461151.1	tBLASTn	*D. melanogaster*	0.00[b]
D. biarmipies[a]	KB462460.1	tBLASTn	*D. melanogaster*	0.00[b]
D. ficusphila[a]	KB457516.1	tBLASTn	*D. melanogaster*	0.00[b]
D. elegans[a]	KB458548.1	tBLASTn	*D. melanogaster*	0.00[b]
D. rhopaloa[a]	KB450382.1	tBLASTn	*D. melanogaster*	6.27E-196
D. bipectinata[a]	KB464224.1	tBLASTn	*D. melanogaster*	1.99E-154
D. ananassae[a]	GF13305	tBLASTn	*D. melanogaster*	4.28E-182
D. persimilis[a]	GL11285	tBLASTn	*D. melanogaster*	1.43E-157
D. miranda[a]	CM001519.2	tBLASTn	*D. melanogaster*	4.16E-92
D. pseudoobscura[a]	GA16191	tBLASTn	*D. melanogaster*	6.51E-160
D. willistoni[a]	GK19556	tBLASTn	*D. melanogaster*	2.72E-150
D. mojavensis[a]	GI21123	tBLASTn	*D. melanogaster*	2.30E-141
D. virilis[a]	GJ20970	tBLASTn	*D. melanogaster*	9.56E-133
D. grimshawi[a]	GH23052	tBLASTn	*D. melanogaster*	2.25E-148
B. cucurbitae	XP_011176821.1	BLASTp	*D. melanogaster*	1E-43
B. dorsalis	XP_011211900.1	BLASTp	*D. melanogaster*	3E-37
C. capitata	XP_004523724.1	BLASTp	*D. melanogaster*	2E-34
M. domestica	XP_011296419.1	BLASTp	*D. melanogaster*	9E-21
G. morsitans morsitans	CCAG010006519.1	BLASTp	*M. domestica*	1.08E-46

[a] The ortholog sequence used in the molecular evolutionary analyses

[b] E-values < E-200 were considered zero

Table S4: C(2)M orthologs confirmed by reciprocal BLAST back to the *D. melanogaster* genome

Species	Accession Number	Method	Seed	E-value
D. melanogaster[a]	CG4249	tBLASTn		0.00[b]
D. simulans[a]	GD24050	tBLASTn	*D. melanogaster*	0.00[b]
D. mauritiana[a]	NA	tBLASTn	*D. melanogaster*	0.00[b]
D. sechellia[a]	GM18665	tBLASTn	*D. melanogaster*	0.00[b]
D. yakuba[a]	GE21442	tBLASTn	*D. melanogaster*	0.00[b]
D. erecta[a]	GG25201	tBLASTn	*D. melanogaster*	0.00[b]
D. eugracilis[a]	KB464450.1	tBLASTn	*D. melanogaster*	0.00[b]
D. takahashii[a]	KB461686.1	tBLASTn	*D. melanogaster*	0.00[b]
D. biarmipies[a]	KB462833.1	tBLASTn	*D. melanogaster*	0.00[b]
D. ficusphila[a]	KB457528.1	tBLASTn	*D. melanogaster*	5.44E-180
D. elegans[a]	KB458274.1	tBLASTn	*D. melanogaster*	0.00[b]
D. rhopaloa[a]	KB451894.1	tBLASTn	*D. melanogaster*	0.00[b]
D. bipectinata[a]	KB464241.1	tBLASTn	*D. melanogaster*	4.70E-97
D. ananassae[a]	GF14579	tBLASTn	*D. melanogaster*	9.62E-91
D. persimilis[a]	GL16321	tBLASTn	*D. melanogaster*	7.09E-94
D. miranda[a]	CM001520.2	tBLASTn	*D. melanogaster*	7.84E-96
D. pseudoobscura[a]	GA18058	tBLASTn	*D. melanogaster*	6.29E-59
D. willistoni[a]	GK23985/partial	tBLASTn	*D. melanogaster*	4.25E-20
D. mojavensis[a]	GI17074	tBLASTn	*D. melanogaster*	2.80E-39
D. virilis[a]	GJ16321	tBLASTn	*D. melanogaster*	4.49E-23
D. grimshawi[a]	GH10313	tBLASTn	*D. melanogaster*	2.60E-23
B. cucurbitae	XP_011190022.1	BLASTp	*D. melanogaster*	6E-03
M. domestica	XP_011292775.1	BLASTp	*D. melanogaster*	1E-04
B. dorsalis	XP_011207064.1	BLASTp	*D. melanogaster*	0.034
C. capitata	XP_004523724.1	BLASTp	*D. melanogaster*	2E-34
M. domestica	XP_011292775.1	BLASTp	*D. melanogaster*	9E-21
G. morsitans morsitans	CCAG010009633.1	BLASTp	*M. domestica*	1.08E-46

[a] The ortholog sequence used in the molecular evolutionary analyses

[b] E-values < E-200 were considered zero

Table S5: C(3)G orthologs confirmed by reciprocal BLAST back to the *D. melanogaster* genome (E-values <E-200 were considered 0).

Species	Accession Number	Method	Seed	E-value
D. melanogaster[a]	CG17604	tBLASTn		0.00[b]
D. simulans[a]	GD20329	tBLASTn	*D. melanogaster*	0.00[b]
D. mauritiana[a]	NA	tBLASTn	*D. melanogaster*	0.00[b]
D. sechellia[a]	GM25754	tBLASTn	*D. melanogaster*	0.00[b]
D. yakuba[a]	GE26360	tBLASTn	*D. melanogaster*	0.00[b]
D. erecta[a]	GG20377	tBLASTn	*D. melanogaster*	0.00[b]
	GG20388	tBLASTn	*D. melanogaster*	0.00[b]
D. eugracilis[a]	KB465333.1	tBLASTn	*D. melanogaster*	0.00[b]
D. takahashii[a]	KB461113.1	tBLASTn	*D. melanogaster*	0.00[b]
D. biarmipies[a]	KB462598.1	tBLASTn	*D. melanogaster*	0.00[b]
D. ficusphila[a]	KB457030.1	tBLASTn	*D. melanogaster*	7.06E-170
D. elegans[a]	KB458458.1	tBLASTn	*D. melanogaster*	3.04E-166
D. rhopaloa[a]	KB448329.1	tBLASTn	*D. melanogaster*	4.97E-192
D. bipectinata[a]	KB464131.1	tBLASTn	*D. melanogaster*	1.29E-02
D. ananassae[a]	GF26923	tBLASTn	*D. melanogaster*	4.44E-22
D. persimilis[a]	GL23692	tBLASTn	*D. melanogaster*	1.32E-02
D. miranda[a]	CM001528.2	tBLASTn	*D. melanogaster*	6.59E-03
D. pseudoobscura[a]	GA26705	tBLASTn	*D. melanogaster*	3.51E-02
D. willistoni	GK10347	BLASTp	*D. melanogaster*	3.95E-05
D. mojavensis	GI14995	BLASTp	*D. virilis*	1.95E-77
D. virilis	GJ15351	BLASTp	*D. melanogaster*	4.15E-05
D. grimshawi	GH12738	BLASTp	*D. virilis*	2.04E-64
B. cucurbitae	-	-	-	-
M. domestica	-	-	-	-
B. dorsalis	-	-	-	-
C. capitata	-	-	-	-
M. domestica	-	-	-	-
G. morsitans morsitans	-	-	-	-

[a] The ortholog sequence used in the molecular evolutionary analyses

[b] E-values < E-200 were considered zero

Table S6: Corolla orthologs confirmed by reciprocal BLAST back to the *D. melanogaster* genome.

Species	Accession Number	Method	Seed	E-value
D. melanogaster[a]	CG8316	tBLASTn		0.00[b]
D. simulans[a]	GD17351	tBLASTn	*D. melanogaster*	0.00[b]
D. mauritiana[a]	NA	tBLASTn	*D. melanogaster*	0.00[b]
D. sechellia[a]	GM13305	tBLASTn	*D. melanogaster*	0.00[b]
D. yakuba[a]	GE15583	tBLASTn	*D. melanogaster*	2.43E-144
D. erecta[a]	GG18173	tBLASTn	*D. melanogaster*	1.36E-129
D. eugracilis[a]	AFPQ02005309.1	tBLASTn	*D. melanogaster*	3.70E-150
D. takahashii[a]	KB461135.1	tBLASTn	*D. melanogaster*	3.56E-42
D. biarmipies[a]	KB462463.1	tBLASTn	*D. melanogaster*	1.09E-45
D. ficusphila[a]	KB457527.1	tBLASTn	*D. melanogaster*	2.19E-37
D. elegans[a]	KB458387.1	tBLASTn	*D. melanogaster*	2.13E-31
D. rhopaloa[a]	KB451800.1	tBLASTn	*D. melanogaster*	3.98E-62
D. bipectinata	AFFE01006525.1	tBLASTn	*D. ananassae*	0.00[b]
D. ananassae	GF22551	BLASTp	*D. melanogaster*	6.36E-07
D. persimilis[a]	GL20392	tBLASTn	*D. miranda*	0.00[b]
D. miranda[a]	CM001516.2	tBLASTn	*D. melanogaster*	1.56E-04
D. pseudoobscura[a]	GA29148	tBLASTn	*D. miranda*	0.00
D. willistoni[a]	GK25608	BLASTp	*D. melanogaster*	1.46E-08
D. mojavensis[a]	GI14631	tBLASTn	*D. melanogaster*	4.12E-16
D. virilis[a]	GJ19282	tBLASTn	*D. melanogaster*	1.59E-08
D. grimshawi[a]	GH12960	tBLASTn	*D. melanogaster*	1.94E-06
B. cucurbitae	-	-	-	-
M. domestica	-	-	-	-
B. dorsalis	-	-	-	-
C. capitata	-	-	-	-
M. domestica	-	-	-	-
G. morsitans morsitans	-	-	-	-

[a] The ortholog sequence used in the molecular evolutionary analyses

[b] E-values < E-200 were considered zero

Table S7: Cona orthologs confirmed by reciprocal BLAST back to the *D. melanogaster* genome.

Species	Accession Number	Method	Seed	E-value
D. melanogaster[a]	CG7676	tBLASTn		0.00[b]
D. simulans[a]	GD19229	tBLASTn	*D. melanogaster*	4.18E-76
D. mauritiana[a]	NA	tBLASTn	*D. melanogaster*	1.27E-77
D. sechellia[a]	GM17867	tBLASTn	*D. melanogaster*	3.47E-77
D. yakuba[a]	GE25535	tBLASTn	*D. melanogaster*	1.08E-102
	GE14694	tBLASTn	*D. melanogaster*	6.46E-101
D. erecta[a]	GG22880	tBLASTn	*D. melanogaster*	1.99E-92
D. eugracilis[a]	KB465338.1	tBLASTn	*D. melanogaster*	1.59E-38
D. takahashii[a]	KB461676.1	tBLASTn	*D. melanogaster*	1.70E-09
D. biarmipies[a]	KB462068.1	tBLASTn	*D. melanogaster*	1.40E-11
D. ficusphila[a]	KB457292.1	tBLASTn	*D. melanogaster*	2.04E-12
D. elegans[a]	KB458397.1	tBLASTn	*D. melanogaster*	6.37E-12
D. rhopaloa[a]	KB452427.1	tBLASTn	*D. melanogaster*	8.61E-15
D. bipectinata	AFFE01007043.1	tBLASTn	*D. ananassae*	7.37E-82
D. ananassae	GF20205	BLASTp	*D. melanogaster*	3.00E-03
D. persimilis[a]	GL20209	tBLASTn	*D. ficusphila*	4.91E-02
D. miranda[a]	CM001516.2	tBLASTn	*D. ficusphila*	8.50E-02
D. pseudoobscura[a]	GA26847	tBLASTn	*D. ficusphila*	4.91E-02
D. willistoni	-	-	-	-
D. mojavensis	GI16118	BLASTp	*D. melanogaster*	6.57E-02
D. virilis	GJ20698	BLASTp	*D. melanogaster*	3.18E-02
D. grimshawi	GH24655	BLASTp	*D. melanogaster*	6.09E-03
B. cucurbitae	-	-	-	-
M. domestica	-	-	-	-
B. dorsalis	-	-	-	-
C. capitata	-	-	-	-
M. domestica	-	-	-	-
G. morsitans morsitans	-	-	-	-

[a] The ortholog sequence used in the molecular evolutionary analyses

[b] E-values < E-200 were considered zero

Table S8: Population parameters of the DGRP and DGPG samples including non-synonymous pairwise diversity (π_N), synonymous pairwise diversity (π_S), and Tajima's D test of neutrality.

		π_N	π_S	Tajima's D	P-value
Ord	N. Carolina	0.00169	0.02240	0.275	0.784
	Africa	0.00193	0.02943	-0.585	0.559
C(2)M	N. Carolina	0.00283	0.01718	0.653	0.514
	Africa	0.00170	0.00973	-0.136	0.892
C(3)G	N. Carolina	0.00224	0.01100	-0.293	0.770
	Africa	0.00230	0.01358	-1.169	0.242
Corolla	N. Carolina	0.00041	0.00091	**-2.055**	**0.040**
	Africa	0.00114	0.00328	**-2.443**	**0.015**
Cona	N. Carolina	0.00383	0.01579	1.023	0.306
	Africa	0.00244	0.02489	-0.527	0.598
Meiosis Means[a]	N. America	0.001	0.013	-	-
	Africa	0.002	0.020	-	-

* Significant *p* values in bold

[a] Mean nucleotide diversity values for all meiotic genes in Anderson *et al*. 2009 included for comparison.
